# Supplementary material for: Developing a new model for patient recruitment in mental health services: a cohort study using Electronic Health Records
Source: BMJ Open. 2014 Dec 2;4(12):e005654. doi: 10.1136/bmjopen-2014-005654 (PMC4256538; doi:10.1136/bmjopen-2014-005654)
Supplement: Author's manuscript [file bmjopen-2014-005654.draft_revisions.pdf]

Realising the potential for clinical research using electronic health records and 'consent for contact' in a mental health context: development and descriptive data

Journal:	*BMJ Open*
Manuscript ID:	bmjopen-2014-005654
Article Type:	Research
Date Submitted by the Author:	12-May-2014

Complete List of Authors:	Callard, Felicity; King's College London (Institute of Psychiatry), ; Durham University,<br>Broadbent, Matthew; South London and Maudsley NHS Fundation Trust,<br>Denis, Mike; South London and Maudsley NHS Fundation Trust,<br>Hotopf, Matthew; King's College London (Institute of Psychiatry),<br>Soncul, Murat; South London and Maudsley NHS Foundation Trust,<br>Wykes, Til; King's College London (Institute of Psychiatry),<br>Lovestone, Simon; King's College London (Institute of Psychiatry), ; South London and Maudsley NHS Foundation Trust and King's College London, NIHR Biomedical Research Centre for Mental Health<br>Stewart, Robert; King's College London, Institute of Psychiatry

**Primary Subject Heading**:	Health informatics
Secondary Subject Heading:	Health informatics, Mental health, Research methods
Keywords:	Health informatics < BIOTECHNOLOGY & BIOINFORMATICS, Information management < BIOTECHNOLOGY & BIOINFORMATICS, Clinical governance < HEALTH SERVICES ADMINISTRATION & MANAGEMENT, MENTAL HEALTH, PSYCHIATRY

SCHOLARONE™ Manuscripts

For peer review only - http://bmjopen.bmj.com/site/about/guidelines.xhtml

Realising the potential for clinical research using electronic health records and 'consent for contact' in a mental health context: development and descriptive data

Authors:

Felicity Callard, Matthew Broadbent, Mike Denis, Matthew Hotopf, Murat Soncul, Til Wykes, Simon Lovestone, Robert Stewart

Dr Felicity Callard, PhD*, King's College London, Service User Research Enterprise, Box P034, Institute of Psychiatry, De Crespigny Park, London SE5 8AF

Visiting Researcher, King's College London, Institute of Psychiatry

Mr Matthew Broadbent, MA, King's College London, SLaM Biomedical Research Centre Nucleus, Institute of Psychiatry, Box P092, De Crespigny Park, London SE5 8AF

Head of BRC Clinical Informatics, NIHR Biomedical Research Centre for Mental Health

Mr Mike Denis, Leonard House, 7 Newman Road, Bromley, BR1 1RJ

Director of Information Strategy, South London and Maudsley NHS Foundation Trust

Prof Matthew Hotopf, PhD, King's College London, Department of Psychological Medicine, Institute of Psychiatry, 10 Cutcombe Rd, London SE5 9RJ

Professor of General Hospital Psychiatry, King's College London, Institute of Psychiatry

Dr Murat Soncul, PhD, Maudsley Hospital, Denmark Hill, London SE5 8AZ

Head of Information Governance, South London and Maudsley NHS Foundation Trust

Prof Til Wykes, PhD, King's College London, Department of Psychology, Institute of Psychiatry, Box P077, De Crespigny Park, London, SE5 8AF

Professor of Clinical Psychology and Rehabilitation

For peer review only

Prof Simon Lovestone, PhD, King's College London, Institute of Psychiatry, Box P070, De Crespigny Park, London SE5 8AF

Professor of Old Age Psychiatry, King's College London, Institute of Psychiatry

Prof Robert Stewart, MD, King's College London, Institute of Psychiatry, Box P063, De Crespigny Park, London SE5 8AF

Professor, King's College London, Institute of Psychiatry

* Corresponding author:

Dr Felicity Callard, Box P034, Institute of Psychiatry, De Crespigny Park, London SE5 8AF, felicity.callard@kcl.ac.uk, +44 7736 720515

For peer review only

For peer review only - http://bmjopen.bmj.com/site/about/guidelines.xhtml

Abstract

Objectives: This research aimed to develop a new model for patient recruitment that harnessed the full potential of electronic health records (EHRs). Gaining access to potential participants' health records to assess their eligibility for studies and allow an approach about participation ('consent to contact') is ethically, legally and technically challenging, given that medical data are usually restricted to the patient's clinical team. The research objective was to design to model for recruitment to overcome some of these challenges as well as reduce the gate-keeper function of clinicians in determining who is appropriate or not to participate in clinical research.

Setting: Large secondary mental health services context, United Kingdom.

Participants: 2,106 patients approached for 'consent for contact'. All patients in different services within the mental health trust are gradually and systematically being approached using the 'consent for contact' model. There are no exclusion criteria.

Primary and secondary outcome measures: Provision of 'consent for contact'.

Results: A new model (the South London and Maudsley NHS Trust Consent for Contact model (SLaM C4C)) for gaining patients' consent to contact them about research possibilities, which is built around a de-identified EHR database. The model allows researchers to contact potential participants directly. Of the 2,106 patients approached by 25 October 2013, nearly three out of every four gave consent for contact (1,560 patients; 74.1%).

Conclusions: The SLaM C4C model offers an effective way of expediting recruitment into health research through the use of EHRs. It reduces the gatekeeper function of clinicians; gives patients greater autonomy in decisions to take part in research; and accelerates the development of a culture of active research participation. More research is needed to assess how many of those giving consent for contact subsequently consent to participate in particular research studies.

Trial registration: N/A

For peer review only

Article Summary

Strengths and limitations of the study

- A new model for gaining patients' consent to contact them about research possibilities, which is built around a robustly de-identified Electronic Health Records (EHR) database
- Currently, 3 out of every 4 patients approached are giving consent to contact using the model
- The model gives patients greater autonomy in decisions about research participation
- The model was developed through extensive service user involvement and consultation
- The study does not examine whether patients who have given consent to contact go on to consent to participation in particular research studies

For peer review only

Introduction

Recruiting patients into health research

One of the most pressing issues for researchers needing to recruit patients is how best to identify who might be suitable for their research, at the same time as adhering to legal and ethical frameworks regarding confidentiality and privacy. Medical data are, in multiple jurisdictions, generally accessible to researchers only with prior patient consent, or if the data are adequately de-identified; this means, in short, that a researcher needs to gain consent from an individual *before* accessing her personal details to adjudicate suitability for the research and before contacting her to ask about potential participation. Access to individuals' medical records is usually restricted to members of the clinical team, who thereby act as intermediaries through which contact between researchers and potential research participants is commonly made. Such a process is, however, often cumbersome and time-consuming: busy clinical teams often do not have the time to assist with recruitment; the alternative path of writing to each patient (e.g. within a general practice) to ask if she would agree to researchers accessing her medical records to assess suitability for a study is no less onerous. Furthermore, should the clinician still be a gate-holder for the patient? Is this not a remnant of medical paternalism, outdated in today's health care system and research context with citizen science and direct-to-consumer research opportunities such as the uBiome[1] project? Clinicians' desire to protect patients can, through 'gate-keeping', end up effectively denying them the opportunity to participate in research.[2] Such a problem is particularly pertinent within a mental health context.

Amidst wide concern about difficulties in recruiting to clinical studies,[3] there are calls to transform the culture of patient participation in health research (e.g. US National Institutes of Health (NIH) Road Map,[4] and England's National Health Service (NHS) Constitution).[5] The influential 2008 Data Sharing Review in the United Kingdom recommended that 'The NHS should develop a system to allow approved researchers to work with healthcare providers to identify potential patients, who may then be approached to take part in clinical studies for which consent is needed'.[6] There appears,

For peer review only - http://bmjopen.bmj.com/site/about/guidelines.xhtml

however, to be a reluctance to mandate legislatively a move away from the current model in which researchers require consent before accessing medical records.

Meanwhile, quantitative and qualitative studies in a number of countries have indicated that significant proportions of the public are cautious about giving researchers access to their medical records for recruitment purposes without prior consent, even as the public remains supportive of health research.[7-9] A recent mass public engagement exercise (6,000 people) in England, for example, found that only 34 per cent of adults and 10 per cent of young people agreed with extending the range of people with direct access to patient records to assist in recruitment to clinical trials.[10] Given this and the current legislative environment, it is likely that in the UK, at least, the current principles surrounding consent (i.e. that researchers are not able to access a patient's records without consent unless they are a member of the patient's clinical team) will continue to underpin research governance for some time. There is therefore an urgent scientific need to find ways of negotiating this governance environment that would allow more efficient identification of, and better engagement with, potential research participants, and would empower patients or service users to make their own decisions regarding which, if any, research projects to learn more about.[11-13]

Consent for contact

One response to this challenge has been the formulation of what has, in the last decade, come to be termed 'consent for contact' or 'consent to consent'. This, as the Academy of Medical Sciences puts it, comprises a 'mechanism … for individuals to give *generic consent to be contacted about suitable research opportunities*, before considering whether they consent to take part in a specific study' [italics added].[14] While such generic consent is appealing for researchers, its operationalization poses a number of ethical, sociological, governance-related and technical questions (see Figure 1). There is growing interest in how EHRs might be used in this regard. To date, the use of EHRs for recruitment has relied on mechanisms through which a member of the clinical team of the patient identified – via pseudonymised searching – as potentially eligible for the study is alerted and invited to contact that patient. Such use therefore currently maintains the

For peer review only - http://bmjopen.bmj.com/site/about/guidelines.xhtml

distinction between those designing the studies and those recruiting into the studies.[15-19]

Insert Figure 1 near here

That many of the questions surrounding the use of EHRs for research remain unresolved, at both a conceptual and an empirical level, is demonstrated by the number of medical, bioethical and governance-oriented bodies currently reflecting on them.[14,20-22] Our paper takes these discussions forward through focusing on an empirical advance: the model of consent for contact developed by the South London & Maudsley (SLaM) NHS Foundation Trust (hereafter: SLaM C4C). For whatever one's position regarding the merits or demerits of 'consent for contact', it is commonly agreed that 'further work is needed to provide guidance and models to enable appropriate access and identification of patients for research'.[23] SLaM C4C was endorsed by the UK's Information Governance Review in 2013 as an exemplar of 'an approach that allows appropriate individuals to be identified and approached to take part, without giving researchers direct access to identifiable information before consent is obtained'.[21]

In the paper we present: (i) the specifics of SLaM C4C, which we believe to be the first successful implementation of consent for contact which both harnesses the potential of de-identified electronic health records to expedite recruitment to research, and allows researchers to contact potential participants directly; (ii) descriptive statistics on how SLaM C4C is being received by patients.

Methods

Ethics committees approvals The SLaM C4C model was reviewed by the National Information Governance Board for Health and Social Care (NIGB) Ethics and Confidentiality committee; the committee considered the model 'an elegant solution to the issue of participant recruitment' and stated that it 'strongly endorse[s] this approach to

For peer review only

participant recruitment'. The model was approved by a Research Ethics Committee (REC), as well as by the SLaM Caldicott Guardian and the SLaM Trust Executive. The Clinical Record Interactive Search (CRIS), around which the model is built, was approved for use by a Research Ethics Committee; its security model (which comprises both technical and procedural elements) was approved by the SLaM Trust Caldicott Guardian, and signed off by the Trust Executive. Full details of all approvals are available as required.

The SLaM C4C model

SLaM C4C is designed to enhance patients' access to opportunities to participate in research projects of interest to them (England's NHS Constitution pledges 'to inform [patients] of research studies in which [they] may be eligible to participate').[5] It also enables researchers (who have undergone rigorous approval procedures) to identify and approach potentially eligible people. The model comprises technical and procedural elements built into the EHR case register at the NIHR Specialist Biomedical Research Centre for Mental Health at the South London & Maudsley (SLaM) NHS Foundation Trust (hereafter: SLaM BRC)). It was designed for use across SLaM, which is one of the largest mental health care providers in Europe, serving a local population of 1.2 million people, and including inpatient wards, outpatient and community services. Prior to this initiative, recruitment to clinical research in SLaM relied on the traditional system of researchers finding clinicians who were willing to identify and approach potential participants. Such nurse, medical and other clinicians may have little or no training in research and scant knowledge of any particular research programme; or they may themselves be researchers. This, together with clinical pressures and conflicting demands on time result at best in the possibility of biased recruitment and, at worse, limit recruitment to research and obstruct patients from making decisions about participation.

The model is embedded within a search and database system (the Clinical Record Interactive Search [CRIS]), which reads and extracts information from SLaM's EHRs, removes identifiers and makes this available to researchers in de-identified format in standard analysis packages (e.g. Stata, Excel). Data consist of all clinical records on

For peer review only - http://bmjopen.bmj.com/site/about/guidelines.xhtml

SLaM patients (unless they have requested to opt out from the register) and are searchable as both structured and free text. There are currently over 220,000 cases on the database, which increases by approximately 20,000 per year. We have described the development and characteristics of CRIS elsewhere[24]; there is also published research based on analyses of CRIS data.[25] Here, we present the features of CRIS that allow research participant recruitment.

Reverse search

The case register was initially approved for use as a de-identified database whose data are searchable without consent by appropriately vetted researchers. The evaluation of this de-identification procedure demonstrated that CRIS effectively ensures patient anonymity at the same time as maximizes data (free text and structured text) that are available for research.[26] The register's technical architecture included, additionally, the potential for reverse search: allowing the identification of patients who meet given characteristics (which can be defined using structured and free text), and thereby the possibility of using CRIS to identify and approach potential participants on the basis of prior consent by individual patients or, for children or adults lacking capacity, an appropriate proxy. In effect, such a mechanism allows for the creation within the case register of a database of people ('the recruitment database') who have provided prior consent to be contacted and whose full – but de-identified – clinical records will be available to a researcher (a 'recruiter') in order to search for inclusion/exclusion criteria for specific, ethically approved studies. The researcher, once s/he has identified eligible potential participants, can then be given identifiers to access the source EHRs and approach patients about participation in that particular study (see Figure 2).

Insert Figure 2 near here

We have developed robust procedural mechanisms to address legal and ethical requirements and to complement this process and technical design, which we briefly outline below.

For peer review only - http://bmjopen.bmj.com/site/about/guidelines.xhtml

Acquiring and recording consent for contact

- The consent process for participation in the recruitment database is conducted entirely by the patient's clinical team, most commonly by the patient's care coordinator. Clinical staff are trained in research governance (particularly regarding consent and the assessment of mental capacity to provide consent) and the specific process of acquiring 'consent to contact'. The process, supported by an information sheet (see Appendices 1a–c), emphasises that patients are not being asked for consent to participate in any particular study – simply to being contacted in the future by researchers about potential participation in specified research projects based on information in their full SLaM EHR. The clinician explains, as required, more about what research is and what it might entail; what tends to be recorded in an EHR; and reminds patient that, if she consents, she might be asked to participate in research on different topics from those relating to her current treatment.
- For patients who are children, or adults lacking capacity (where the lack of capacity is believed to be permanent), proxy assent is sought from a close friend or relative (with parental responsibility in the case of children) (see Appendices 1b and 1c). The model is attentive to the mental health context for which it was designed: where mental capacity is thought to be temporarily absent, the clinical team does not ask for consent, but aims to approach the patient at a later time in accordance with the second principle of the Mental Capacity Act.[27]
- The approach made by a clinician requesting consent to contact is tailored to the clinical context – for example, the request may be delayed for a distressed patient admitted in a crisis.
- A research participation form has been created as an additional window in the source clinical records system (see Figure 3). This includes:
    - Whether a C4C discussion has taken place with the patient (or a named proxy);
    - Whether the patient (or proxy) gave consent or not;
    - The name of the clinical staff member discussing C4C and the date discussed;

For peer review only - http://bmjopen.bmj.com/site/about/guidelines.xhtml

- A free text box to record any further information. Notably, patients are encouraged to identify any particular preferences regarding research opportunities – both those that they would be particularly interested in, and kinds of research, or research topics, that they would not want to be contacted about.

Insert Figure 3 near here

- For patients who have consented to being contacted, the research participation form is also used to record all subsequent contacts made by researchers, and all projects the patient is participating in, has participated in, and/or has declined to participate in. If a patient withdraws consent to be contacted then the technical ability to link to his or her EHR ID will be prevented.
- The model acknowledges that patients might not be aware, at the point of giving consent to participating in the recruitment database, what information will later be contained in their medical record. All consents are revisited with the participant at the point of discharge from all SLaM services or else are deemed to have expired.

Identifying and contacting potential research participants

- In order to become a 'recruiter' – i.e. have the ability to access CRIS for approaching potential research participants – the researcher must have a substantive or honorary contract with SLaM and be an employee of one of the organisations forming King's Health Partners, the Academic Health Sciences Centre (AHSC) of which SLaM is a member. Use of CRIS for all forms of research is logged and audited and misuse would result in disciplinary action.
- Recruiters use de-identified data on CRIS to identify potential recruits. In CRIS itself, records are identified by a locally generated pseudonym (the BRC ID). This pseudonym is linked to the EHR ID number in the building of the CRIS data

For peer review only - http://bmjopen.bmj.com/site/about/guidelines.xhtml

repository. Research users of CRIS are unable to access the link between BRC ID and EHR ID (Figure 2), which maintains the integrity of CRIS as a de-identified database (Figure 1).

- An Oversight Committee (which is chaired by a mental health service user, and which includes committee members who do not use CRIS for research as well as experts on ethics, research and information governance) manages all access to, and monitors all use of, CRIS, and reports to the Trust Caldicott Committee. The Oversight Committee uses ethical, legal and scientific criteria to adjudicate such access and use. Applications to use CRIS to identify potential recruits are considered only for projects that have specific ethics and research governance approval. In the UK, acquiring this approval necessitates specifying how exactly patients will be recruited. This provides an additional layer of oversight, which helps to ensure that appropriate modes of approach are made to patients who have given 'consent to contact' when the topic of research is sensitive (e.g. researchers might be required to talk to the treating clinician before contacting the patient).
- The recruiter submits the pseudonymised BRC ID of potential recruits s/he wishes to contact to a trusted third party (TTP) appointed and monitored by the Oversight Committee.
- The TTP has project-specific access to the database linking the BRC IDs with the source EHR IDs. Technical specifications within the system ensure that CRIS cannot return the EHR ID of any patient who has not given consent for contact. Following reverse search, the TTP passes the EHR IDs of the potential recruits that have given consent back to the recruiter.
- The recruiter, through the SLaM EHR, is then able to identify and contact the patient to discuss participation in the project. Safeguards include a time limit on when the recruiter may contact patients about recruitment, and the requirement that recruiters inform each patient's care coordinator by email that they will be contacting that patient in a few days' time (to allow the opportunity for the care coordinator to suggest, for example, that an approach at the current time might not be advisable).

For peer review only

- The Oversight Committee monitors the research participation forms to ascertain if approaches for research participation or actual research participation appears excessive. What would constitute 'excessive' is an ethical, sociological and scientific question: what might be excessive for certain individual patients, or groups of patients, might not be for others; participating in multiple surveys might be adjudicated differently from participation in several clinical trials in a short time frame. The oversight committee is formalizing guidelines to adjudicate and respond to putatively 'excessive' research participation. Options for action include contacting the patient or clinical team about whether the patient continues to be willing to be approached about further research projects during the course of the current projects in which they are participating.

Involving and engaging stakeholders

There is increasing acknowledgement of the importance of engaging stakeholders and the public in designing ethical and technically robust procedures to guide the use of EHRs.[8,10,11,28] Mental health service users made significant contributions to the development of our model. The Patient and Carer Participation Theme within the SLaM BRC took a central role in developing the model; the Oversight Committee is chaired by a mental health service user; and consultation and engagement with service user groups have taken place throughout, in line with our BRC's model of involving service users at all stages of translational research.[29] An annual newsletter is sent to all persons in the recruitment database summarising key findings arising from the SLaM BRC, in addition to reminding people of contact details for the database (for example, if they wish later to withdraw). (See Figure 4 for a summary of key features of the SLaM C4C model.)

Insert Figure 4 near here

Results

Summary statistics from the implementation of SLaM C4C

For peer review only - http://bmjopen.bmj.com/site/about/guidelines.xhtml

SLaM C4C started to be implemented across the South London & Maudsley NHS Trust in May 2012, and the aim is to implement C4C across the entire Trust, which serves a total of approximately 35,000 active patients, split across seven Clinical Academic Groups (CAGs). All patients are gradually being approached and asked whether they will provide 'consent for contact'. There are no exclusion criteria. Implementation commenced in services where there was already support for and/or enthusiasm about C4C. The figures from 25 October 2013 show that a total of 2,106 patients had been approached, of whom 1,560 had given consent and 546 had not: a 74.1% consent rate. Table 1 presents descriptive statistics, from October 2013, of those approached to give consent for contact, in relation to gender, ethnicity and age.

Insert Table 1 near here

Table 1: Descriptive data regarding patients approached for SLaM C4C

	N total	N consenting	Consent rate (%)
Gender			
**Male**	1078	844	78.3
**Female**	1028	716	69.6
Self-assigned Ethnicity (amalgamated)			
**White**	1228	894	72.8
**Caribbean, African or Any other Black Background**	496	360	72.6
**Not stated**	205	168	82.0
**Other**	100	78	78.0
**Indian, Pakistani, Bangladeshi or Any other Asian Background**	77	60	77.9
Age*			
**0 to 19**	841	667	79.3
**20 to 29**	182	165	90.7
**30 to 44**	232	213	91.8
**45 to 74**	370	283	76.5
**75 +**	481	232	48.2

For peer review only - http://bmjopen.bmj.com/site/about/guidelines.xhtml

Data captured on 25 October 2013.

* NB Age distribution is not representative of the Trust as a whole, since implementation of SLaM C4C to date has significantly focused on Mental Health of Older Adults Services, and Child and Adolescent Mental Health Services

Discussion

Initial data suggest that the SLaM C4C model is capable of accelerating the development of a culture of active research participation that is founded on the ethical and effective use of electronic health records. The model has been successfully developed with significant service user involvement, has received necessary approval and endorsement from all relevant governance bodies, and is currently resulting in almost three of every four patients approached agreeing to join the recruitment database. Public surveys and bioethical analyses commonly indicate that mental health is a sensitive domain in relation to EHRs, in light of concerns about potential stigma and discrimination.[28,30,31] The current consent figures in this secondary mental health services context are therefore encouraging vis-à-vis the model's transferability elsewhere.

Preliminary data from the implementation of SLaM C4C raise some interesting lines of investigation. That there are currently lower rates of consent from patients over 75 concurs with other research that indicates that older people are more likely to refuse participation in health research.[32] That there are currently higher rates of consent by men than women parallels other findings indicating that men are more likely to consent than women to a review of their medical records.[31] Variations in consent rates pose questions about possible selection bias in studies using this recruitment database. More research is needed to assess how many of those giving consent for contact subsequently consent to participate in particular research studies.

The model that we have presented adheres to current best practice in recruiting patients[21], most notably as regards ensuring that no identifiable patient information is available to researchers without the patient's consent. The model appears to be effective in its implementation – both in enabling the creation of a recruitment database, and in

For peer review only - http://bmjopen.bmj.com/site/about/guidelines.xhtml

terms of acceptability to patients. We believe the model to be generalizable to other health services contexts that employ EHRs. Our intent when designing it was to guard against the erosion of trust in research – a key risk associated with the use of medical records without consent[30] – both through adhering to consent for contact principles and committing regularly to engage with members of the recruitment database about ongoing research and about their current willingness to be contacted about potential research possibilities. We designed the model to allow patients greater autonomy in decisions to take part in research, through lessening the gatekeeper role of clinicians: evidence from a prospective cohort study and a qualitative process evaluation indicates that such gatekeeper functions can impede equitable access to research.[13,33]

A new culture of research participation?

The use, linkage and further development of large EHR datasets are likely to transform relations between researchers, clinicians, patients and their data. The implementation of SLaM C4C, across a large mental health provider, generates, we believe, effectively a field site or laboratory in which to study these potential transformations. The Oversight Committee regularly captures descriptive data that give broad indications of how implementation of SLaM C4C is proceeding (both as regards patients approached, and as regards the number and type of research studies applying to use SLaM C4C). In addition, SLaM BRC is planning additional research and evaluation studies (see Figure 5 for indicative research studies).

Insert Figure 5 near here

Our BRC's ongoing collection of survey and audit data, as well as planned research studies, comprise a powerful means through which to interrogate and analyse the sociological, ethical, technical and governance-related ramifications of large-scale EHR implementation of consent for contact, in which clinicians no longer provide the primary conduit for patient participation in research.

For peer review only - http://bmjopen.bmj.com/site/about/guidelines.xhtml

Acknowledgements

We thank the NIGB Ethics and Confidentiality Committee for its helpful comments on and input into the final design of the model for recruitment. We also thank the CRIS Oversight Committee and the CRIS Security & Confidentiality Procedures Working Group.

Funding Sources

FC, MB, MH, TW, SL and RS acknowledge financial support from the National Institute for Health Research (NIHR) Specialist Biomedical Research Centre for Mental Health award to the South London and Maudsley NHS Foundation Trust and the Institute of Psychiatry, King's College London. SL and TW acknowledge the NIHR for their Senior Investigator Awards. The development of the SLAM BRC Case Register was funded by a Capital Award from the UK NIHR and is further supported through the BRC Nucleus funded jointly by the Guy's and St Thomas' Trustees and South London and Maudsley Special Trustees. The funders had no role in study design, data collection and analysis, decision to publish, or preparation of the manuscript.

Funding The authors acknowledge financial support from the National Institute for Health Research (NIHR) Specialist Biomedical Research Centre for Mental Health award to the South London and Maudsley NHS Foundation Trust and the Institute of Psychiatry, King's College London. The development of the SLAM BRC Case Register was funded by a Capital Award from the UK NIHR and is further supported through the BRC Nucleus funded jointly by the Guy's and St Thomas' Trustees and South London and Maudsley Special Trustees.

Authors' contributions

The idea for the manuscript was conceived by FC in conversation with RS and SL. FC drafted the manuscript, with substantial text contributions from MB, SL and RS. All authors were involved in critical revision of the manuscript before submission, and approved the final manuscript. All authors developed and/or refined the consent for

For peer review only

For peer review only - http://bmjopen.bmj.com/site/about/guidelines.xhtml

contact model described in the paper. FC is the guarantor. FC is Chair of the CRIS Oversight Committee and was Chair of the Security and Confidentiality Procedures Working Group that developed the security model for CRIS; MB project managed the development of CRIS; MD directs information strategy within SLaM; MH developed the protocol for patients lacking mental capacity; MS is Head of Information Governance and the Caldicott representative at SLaM; TW is Patient and Carer Participation Theme Lead for the Biomedical Research Centre for Mental Health; SL is director of the Biomedical Research Centre for Mental Health; RS led the academic development of CRIS. RS, SL and MH secured funding for the work.

Competing Interests

SL is co-cordinator of the European Medical Information Framework (www.emif.eu), a public private partnership with funding from EU and pharmaceutical companies that seeks to make use of both research and routine clinical data for research.
There are no other conflicts of interest.

Data Sharing Statement

Extra data are available by emailing felicity.callard@kcl.ac.uk

For peer review only

References

1. uBiome. http://ubiome.com [Accessed 2 January 2014].
2. Higginson IJ, Evans CJ, Grande G, Preston N, Morgan M, et al. (2013) Evaluating complex interventions in end of life care: the MORECare statement on good practice generated by a synthesis of transparent expert consultations and systematic reviews. BMC Med 11: 111.
3. Ross S, Grant A, Counsell C, Gillespie W, Russell I, et al. (1999) Barriers to participation in randomised controlled trials: a systematic review. J Clin Epidemiol 52: 1143-1156.
4. Zerhouni EA (2007) Translational research: moving discovery to practice. Clin Pharmacol Ther 81: 126-128.
5. Department of Health (2013) The NHS Constitution for England. Department of Health.
6. Thomas R, Walport M (2008) Data Sharing Review Report. London: Ministry of Justice.
7. Buckley BS, Murphy AW, MacFarlane AE (2011) Public attitudes to the use in research of personal health information from general practitioners' records: a survey of the Irish general public. J Med Ethics 37: 50-55.
8. Armstrong V, Barnett J, Cooper H, Monkman M, Moran-Ellis J, et al. (2007) Public perspectives on the governance of biomedical research: a qualitative study in a deliberative context. London: Wellcome Trust.
9. Willison DJ, Schwartz L, Abelson J, Charles C, Swinton M, et al. (2007) Alternatives to project-specific consent for access to personal information for health research: what is the opinion of the Canadian public? J Am Med Inform Assoc 14: 706-712.
10. new economics foundation (2010) Who sees what: exploring public views on personal electronic health records. London: new economics foundation.
11. Academy of Medical Sciences (2006) Personal data for public good: using health information in medical research. London: The Academy of Medical Sciences.
12. Hewison J, Haines A (2006) Overcoming barriers to recruitment in health research. BMJ 333: 300-302.
13. Knowles RL, Bull C, Wren C, Dezateux C (2011) Ethics, governance and consent in the UK: implications for research into the longer-term outcomes of congenital heart defects. Arch Dis Child 96: 14-20.
14. Academy of Medical Sciences (2011) A new pathway for the regulation and governance of health research. London: The Academy of Medical Sciences.
15. Brooks CJ, Stephens JW, Price DE, Ford DV, Lyons RA, et al. (2009) Use of a patient linked data warehouse to facilitate diabetes trial recruitment from primary care. Prim Care Diabetes 3: 245-248.
16. Embi PJ, Jain A, Clark J, Bizjack S, Hornung R, et al. (2005) Effect of a clinical trial alert system on physician participation in trial recruitment. Arch Intern Med 165: 2272-2277.

For peer review only - http://bmjopen.bmj.com/site/about/guidelines.xhtml

For peer review only

17. McGregor J, Brooks C, Chalasani P, Chukwuma J, Hutchings H, et al. (2010) The Health Informatics Trial Enhancement Project (HITE): Using routinely collected primary care data to identify potential participants for a depression trial. Trials 11: 39.
18. Ainsworth J, Buchan I (2009) Preserving consent-for-consent with feasibility-assessment and recruitment in clinical studies: FARSITE architecture. Stud Health Technol Inform 147: 137-148.
19. Stuardi T, Cox H, Torgerson DJ (2011) Database recruitment: a solution to poor recruitment in randomized trials? Fam Pract 28: 329-333.
20. Nuffield Council on Bioethics (2013) New Working Party on Biodata. Nuffield Council on Bioethics.
21. Caldicott F (2013) Information: to share or not to share?: the information governance review. London: Department of Health.
22. Great Britain. Department of H Information: to share or not to share?: Government response to the Caldicott Review. In: Great Britain. Department of H, editor: Department of Health, 2013.
23. Health Research Authority (2013) The HRA welcomes the publication of Dame Fiona Caldicott's Information Governance Review.
24. Stewart R, Soremekun M, Perera G, Broadbent M, Callard F, et al. (2009) The South London and Maudsley NHS Foundation Trust Biomedical Research Centre (SLAM BRC) case register: development and descriptive data. BMC Psychiatry 9: 51.
25. Chang CK, Hayes RD, Broadbent M, Fernandes AC, Lee W, et al. (2010) All-cause mortality among people with serious mental illness (SMI), substance use disorders, and depressive disorders in southeast London: a cohort study. BMC Psychiatry 10: 77.
26. Fernandes AC, Cloete D, Broadbent MT, Hayes RD, Chang CK, et al. (2013) Development and evaluation of a de-identification procedure for a case register sourced from mental health electronic records. BMC Med Inform Decis Mak 13: 71.
27. (2007) Mental Capacity Act 2005 : code of practice. London: TSO.
28. Ipsos MORI (2007) The use of personal health information in medical research: general public consultation. Final report. London: Medical Research Council.
29. Callard F, Rose D, Wykes T (2012) Close to the bench as well as at the bedside: involving service users in all phases of translational research. Health Expect 15: 389-400.
30. Rothstein MA (2010) Is deidentification sufficient to protect health privacy in research? Am J Bioeth 10: 3-11.
31. Hill EM, Turner EL, Martin RM, Donovan JL (2013) "Let's get the best quality research we can": public awareness and acceptance of consent to use existing data in health research: a systematic review and qualitative study. BMC Med Res Methodol 13: 72.
32. Williams B, Irvine L, McGinnis AR, McMurdo ME, Crombie IK (2007) When "no" might not quite mean "no"; the importance of informed and meaningful non-consent: results from a survey of individuals refusing participation in a health-related research project. BMC Health Serv Res 7: 59.

For peer review only

33. Patterson S, Kramo K, Soteriou T, Crawford MJ (2010) The great divide: a qualitative investigation of factors influencing researcher access to potential randomised controlled trial participants in mental health settings. J Ment Health 19: 532-541.

For peer review only

For peer review only - http://bmjopen.bmj.com/site/about/guidelines.xhtml

Figure 1: 'Consent for contact': sociological, ethical and technical issues

- Does 'consent for contact' reshape relationships between treating clinicians, patients and researchers – in that the traditional role of clinician – as patient advocate and/or paternalistic patient protector – in relation to the researcher is downgraded in the emergence of a new kind of compact between patient and researcher?
- Does patients' giving of generic consent to be contacted affect how they subsequently respond to invitations to participate in specific research projects (i.e. do they feel more of an onus to give consent here, too, having given consent once already)?
- Does 'consent for contact' encourage an assumption that willingness to participate in health research is a moral obligation[34] – and if so, what are the ethical, clinical and sociological implications?
- How can electronic health records be best used in developing consent for contact procedures? How should their use navigate complex questions regarding control, ownership and use of such data in relation to consent, authorization, and safe-keeping?[21]

For peer review only

Figure 2: The SLaM 'consent for contact' model

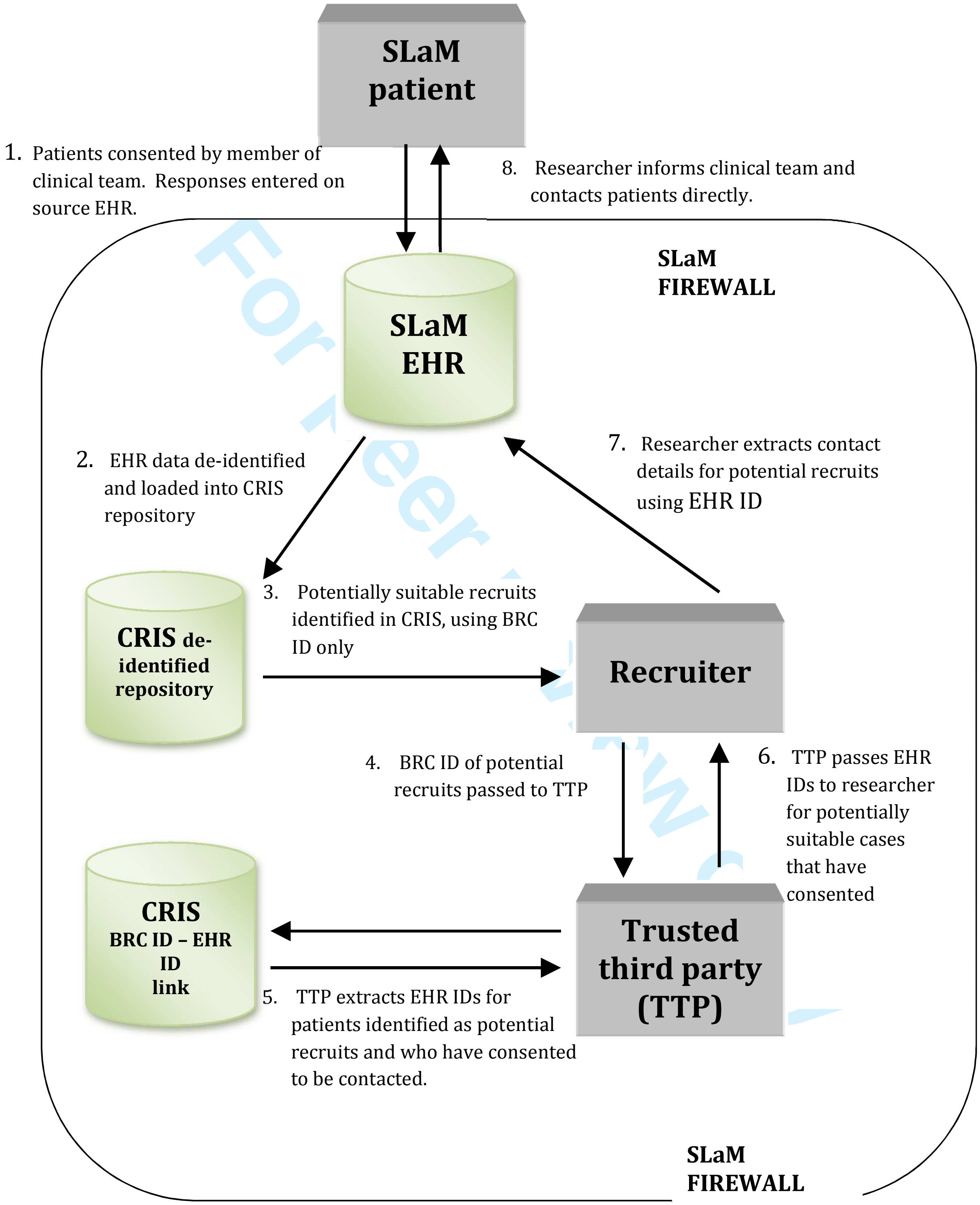

Figure 3: Key features of the SLaM C4C model

- Extensive service user involvement throughout model's development.
- The initial contact is made by a member of the patient's clinical care team.
- Researchers gain access to identifiable information only for patients who have given prior consent.
- All recruiting researchers have undergone extensive vetting procedures, and are employed on contracts that have the same level of duties and responsibilities (and same potential penalties re disciplinary action and dismissal) as those with substantive clinical contracts.
- Patients revisit their consent to be part of the recruitment database on discharge from the service.
- Regular (at least yearly) engagement with those consenting to be part of the recruitment database – about ongoing research studies, as well as reminders about their consenting to be part of the recruitment database and details on how to withdraw consent.

For peer review only

Figure 4: Screen shot of the SLaM C4C Patient Participation Form in the original source EHR

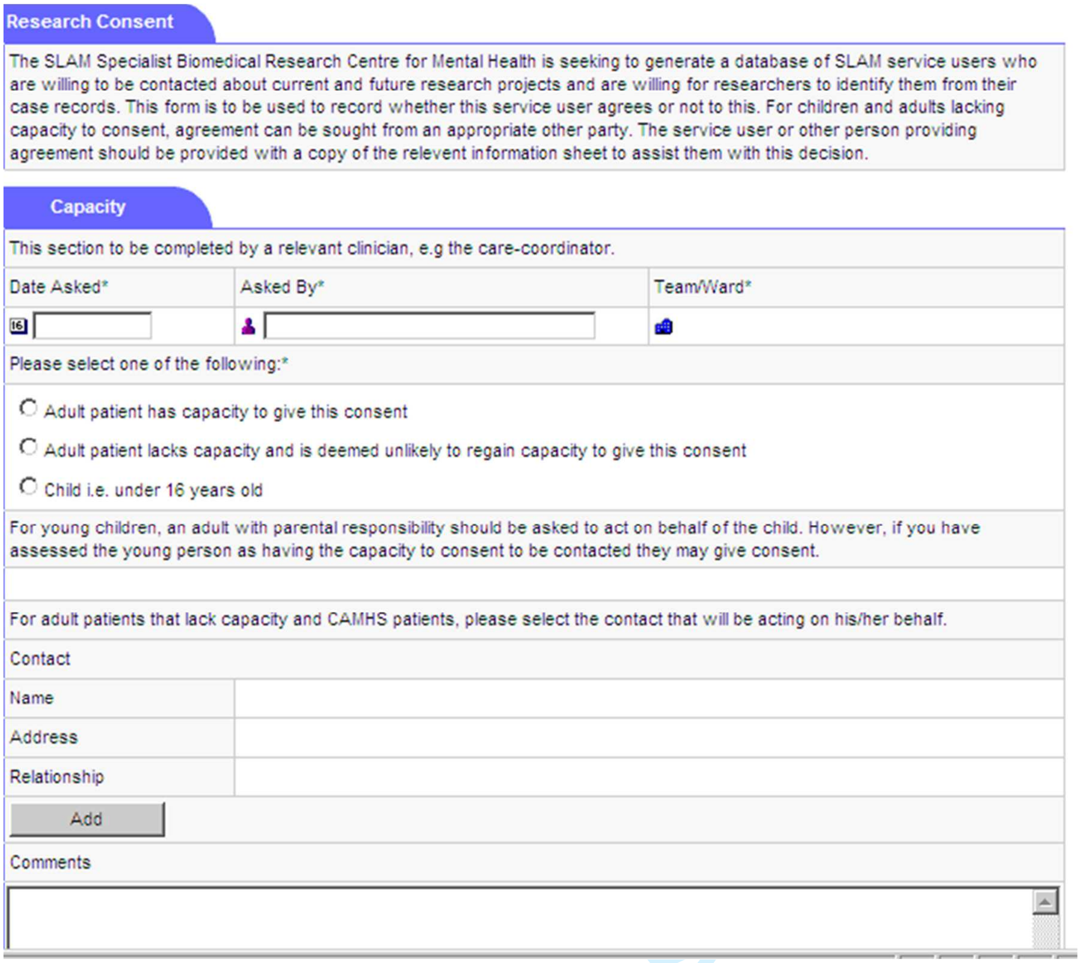

Research Consent

The SLAM Specialist Biomedical Research Centre for Mental Health is seeking to generate a database of SLAM service users who are willing to be contacted about current and future research projects and are willing for researchers to identify them from their case records. This form is to be used to record whether this service user agrees or not to this. For children and adults lacking capacity to consent, agreement can be sought from an appropriate other party. The service user or other person providing agreement should be provided with a copy of the relevent information sheet to assist them with this decision.

Capacity

This section to be completed by a relevant clinician, e.g the care-coordinator.

Date Asked*	Asked By*	Team/Ward*

Please select one of the following:*

- Adult patient has capacity to give this consent
- Adult patient lacks capacity and is deemed unlikely to regain capacity to give this consent
- Child i.e. under 16 years old

For young children, an adult with parental responsibility should be asked to act on behalf of the child. However, if you have assessed the young person as having the capacity to consent to be contacted they may give consent.

For adult patients that lack capacity and CAMHS patients, please select the contact that will be acting on his/her behalf.

Contact

Name	
Address	
Relationship	

Add

Comments

Permission To Contact

This section to be completed by a relevant clinician, e.g the care-coordinator.

Date Asked*	Asked By*	Team/Ward*

Following discussion with the patient or the parent/carer if the patient lacks capacity, please confirm if he/she consents to both the following:

1. I agree to be contacted by a researcher offering the opportunity to take part in relevent research projects if they think I may be a suitable participant.
2. If I agree to be contacted as a potential participant in relevent research projects, I understand that sections of the clinical record may be looked at by responsibile individuals from a reserach team to see if I am eligible to be approached about particular projects. I give permission for these individuals to have access to my records for this reason and to be approached to explain the relevant projects(s).

Information leaflet provided	Permission asked at	Response
☐ Yes	- Please Select -	○ Yes ○ No

Add

Comments

Approaches and Participation

This section should be completed by BRC researchers
It should be used to record all occasions where the patient was invited to participate in a potentially suitable project.
An end date should be entered at the start if known, e.g based on study recruitment period length or length of study ethics approval.

Date Asked	Asked By	Project	Ethics Approval Ref	Response	End Date
				○ Yes ○ No	

Add

w only

Figure 5: Research and evaluation questions raised by the SLaM C4C model

- *Conveying consent for contact to patients.* One current study is using focus groups and reiterative methods to ask: What kinds of explanations of consent for contact are most clearly comprehensible to patients?
- *Patients' experience of consent for contact.* How do patients interpret the process of giving (or refusing) generic consent to access medical records? Does the SLaM C4C model affect their sense of autonomy vis-à-vis decisions about participating in health research? How often do those who give consent for contact go on to participate in research?
- *Researchers' experience consent for contact.* Does it facilitate recruitment (in terms of ease of recruiting, time to reach recruitment targets)? Does the possibility of being able to use free-text in CRIS to identify and cluster patients change how studies are designed?
- *Clinicians' experience of consent for contact (both those who make the approach for 'consent for contact' and others).* How, practically, do clinicians carry out and record consent for contact discussions with their patients? Does consent for contact alter relationships between clinicians and patients?
- *Differential rates of those consenting.* How and why do rates of consent vary across demographic groups and types of service? Does this produce selection bias in studies recruiting from the database?
- *Consent for contact as a potential mechanism to help facilitate public engagement vis-à-vis participation in health research and broader use of, and trust in, EHRs.*[28,31]

For peer review only

South London and Maudsley NHS
NHS Foundation Trust

Participating in Research: Information for patients

Biomedical Research Centre
Clinical Record Interactive Search

We are looking for people who wish to participate in research. In order to contact you about research projects for which you might be suitable, we would like to ask your permission to review your health records at a future date.

This research is conducted within the Biomedical Research Centre, which is a partnership between South London and Maudsley NHS Foundation Trust (SLaM) and the Institute of Psychiatry at King's College London.

Our aim is to convert research findings into clear improvements in health for people with mental health and related problems. You can help us to do this. Volunteers who take part in research play a crucial role in improving the lives of thousands of people. We are creating a list of people who might be interested in taking part in current or future research projects.

If you agree to be part of this list, our researchers will be able to look at your SLaM records to see if you might be suitable for a research project (for example because of the symptoms you are experiencing or the medication you are taking). If you are potentially suitable for a project, a researcher will contact you to discuss whether you might like to participate.

We are only asking for access to your full clinical record with SLaM. We will not need to see other records.

For peer review only

What type of research is carried out at the Biomedical Research Centre for Mental Health?

We carry out a large amount of research into the causes and consequences of mental health problems, from symptoms occurring in children to those occurring at the end of life. The Biomedical Research Centre particularly focuses on finding better ways to treat mental health problems and improve the lives of those affected. Our research projects have examined common mental health disorders (like anxiety and depression), dementia, psychosis, substance use disorders, and much more. The research you might take part in could involve brain scans, puzzles, blood tests, questionnaires, interviews, computer games, or completion of a week-long diary.

For more information about our research, visit the SLaM website and search for 'BRC': http://www.slam.nhs.uk. Research is the only way to develop better treatments. Nearly all research at some point needs the help of those affected by particular health conditions in order to develop these treatments.

What will happen if I agree to be on the list?

If you agree to be on the list, this simply means that you are allowing our researchers to contact you if they think you might be suitable for a particular research project. It is possible that you may not be contacted at all, and it is possible that you will be contacted about a study which is not directly relevant to the symptoms you have been experiencing. If you agree for your name to be on this list, there is no commitment to take part in any future project.

Our researcher would approach you with information about each individual project, and would give you time to think about participating. You would then be free to agree or disagree without giving a reason for this. All we are asking for now is your permission to review your SLaM health records in the future so that our researchers can contact you about research projects for which you might be suitable.

There may not be a project suitable for you to take part in straightaway, but we will keep people who are on the list updated about the research being done through an annual newsletter.

For peer review only

Can I choose the research projects I would like to be involved in?

Yes. You can specify any types of research project you would be interested in or would prefer not to be contacted about. You can let your care coordinator know about your preferences and these can be recorded on your electronic record.

If you wish to limit the number of times in a year we approach you about research studies, your care coordinator can also include this on your record, as well as any other concerns you might have.

If you are discharged from SLaM in the future, we will ask you again about whether you wish to remain on this list, but otherwise your name will be removed automatically. A decision to withdraw at any time, or a decision not to be put on the list in the first place, will not in any way affect the standard of care you receive.

Does I have to be on the list?

No. It is up to you to decide whether you want to be on the list. This is an ‘opt in’ (rather than an ‘opt out’) system. You do not have to give a reason for not being on the list. If you decide to be on the list, your permission will be recorded on your electronic health record (i.e. on the computer system). We will give you a copy of this information for you to keep.

You are free to withdraw this agreement at any time without giving a reason. If you want to withdraw, you can ask your care coordinator to alter the health record, or contact the list administrator (contact details below).

Will my personal details be kept confidential?

We will comply with UK confidentiality laws to make sure that confidentiality is protected. Only our researchers who have gone through the necessary checks required by law can see personal information. However, when considering this, it is important that you realise that any piece of information recorded in your case record might potentially be used as a basis for contacting you in the future. If you wish to participate but do not wish a particular part of your record to be used in this way then please let your care coordinator know and they can record this preference. If you are interested in finding out more about the list or participating in any of our research you can contact the list administrator by phoning 020 3228 8553 or emailing cris.administrator@kcl.ac.uk

For peer review only

South London and Maudsley NHS
NHS Foundation Trust

Contact SLaM

SLaM switchboard: 020 3228 6000
SLaM website: www.slam.nhs.uk

Patient Advice and Liaison Service (PALS)

PALS is here to listen and support you in whatever way we can. We want your experience at SLaM to be positive. If you are not happy about something at SLaM, we will try to help you. If you decide you want to make a complaint, we can advise you how to do this.

PALS 24hr information line: 0800 731 2864
PALS website: www.slam.nhs.uk/pals
PALS email: pals@slam.nhs.uk

Travel

For the quickest way to plan your journey to a SLaM service try Transport for London's online journey planner - www.tfl.gov.uk/journeyplanner or call 020 7222 1234 for 24hr travel information.

If you would like a large print, audio, Braille or a translated version of this leaflet then please ask us.

only

KING'S HEALTH PARTNERS

Reference: C4C
Date of publication: 1/3/2012
Review date: 1/3/2013

Pioneering better health for all

For peer review only - http://bmjopen.bmj.com/site/about/guidelines.xhtml

South London and Maudsley NHS Foundation Trust

Participating in Research: Information for parents and guardians

Biomedical Research Centre
Clinical Record Interactive Search

We are looking for people who wish to participate in research. In order to contact you about research projects for which your son or daughter might be suitable, we would like to ask your permission to review their health records at a future date.

This research is conducted within the Biomedical Research Centre, which is a partnership between South London and Maudsley NHS Foundation Trust (SLaM) and the Institute of Psychiatry at King's College London.

Our aim is to convert research findings into clear improvements in health for people with mental health and related problems. You can help us to do this. Volunteers who take part in research play a crucial role in improving the lives of thousands of people. We are creating a list of people who might be interested in taking part in current or future research projects.

This includes people receiving SLaM services and we are asking you as the parent (or adult with parental responsibility) whether you agree to the inclusion of your son or daughter on this list.

If you agree for your son or daughter to be part of this list, our researchers will be able to look at their SLaM records to see if they might be suitable for a research project (for example because of the symptoms they are experiencing or the medication they are taking). If they are potentially suitable for a project, a researcher will contact you and your son or daughter to discuss whether they might like to participate.

We are only asking for access to their full clinical record at SLaM. We will not need to see other records.

For peer review only

What type of research is carried out at the Biomedical Research Centre for Mental Health?

We carry out a large amount of research into the causes and consequences of mental health problems, from symptoms occurring in children to those occurring at the end of life. The Biomedical Research Centre particularly focuses on finding better ways to treat mental health problems and improve the lives of those affected. Our research projects have examined common mental health disorders (like anxiety and depression), dementia, psychosis, substance use disorders, and much more. The research your son or daughter might take part in could involve brain scans, puzzles, blood tests, questionnaires, interviews, computer games, or completion of a week-long diary.

For more information about our research, visit the SLaM website and search for 'BRC': http://www.slam.nhs.uk. Research is the only way to develop better treatments. Nearly all research at some point needs the help of those affected by particular health conditions in order to develop these treatments.

For peer review only

What will happen if my son or daughter is on the list?

If you agree to your son or daughter being on the list, and if they do not object themselves, this simply means that you are both allowing our researchers to get in contact if they think your son or daughter might be suitable for a particular research project. It is possible that you may not be contacted at all, and it is possible that you will be contacted about a study which is not directly relevant to the symptoms your son or daughter has been experiencing.

If you agree to your son's or daughter's name being on this list, there is no commitment to take part in any future project. Our researcher would approach you both with information about each individual project, and would give you both time to think about participating. Both of you would then be free to agree or disagree without giving a reason for this.

All we are asking for now is your permission to review this person's SLaM health records in the future so that our researchers can make contact about research projects for which your son or daughter might be suitable.

There may not be a project suitable for your son or daughter to take part in straightaway, but we will keep people who are on the list updated about the research being done through an annual newsletter.

Can we choose the research projects we would like to be involved in?

Yes. Either you can specify any types of research project you will be interested in or would prefer not to be contacted about. You can let the person's care coordinator know about your preferences and these can be recorded on their electronic record.

If either of you wish to limit the number of times in a year we approach you about research studies, their care coordinator can also include this on their record, as well as any other concerns you might have.

If your son or daughter is discharged from SLaM in the future, we will ask you again about whether you wish them to remain on this list, but otherwise their name will be removed automatically. A decision to withdraw at any time, or a decision not to be put on the list in the first place, will not in any way affect the standard of care they receive.

Does my son or daughter have to be on the list?

No. It is up to both of you to decide whether you want to be on the list. This is an 'opt in' (rather than an 'opt out') system. You do not have to give a reason for not being on the list. If both of you decide to be on the list, your permission will be recorded on the person's electronic health record (i.e. on the computer system). We will give you a copy of this information for you to keep.

Either of you are free to withdraw this agreement at any time without giving a reason. If either of you want to withdraw, you can ask your son's or daughter's care coordinator to alter the health record, or contact the list administrator (contact details below).

Will personal details be kept confidential?

We will comply with UK confidentiality laws to make sure that confidentiality is protected. Only our researchers who have gone through the necessary checks required by law can see personal information. However, when considering this, it is important that you realise that any piece of information recorded in you son's or daughter's case record might potentially be used as a basis for making contact in the future. If you wish to participate but do not wish a particular part of their record to be used in this way then please let their care coordinator know and they can record this preference. If you are interested in finding out more about the list or participating in any of our research you can contact the list administrator by phoning 020 3228 8553 or emailing cris.administrator@kcl.ac.uk

For peer review only

South London and Maudsley NHS
NHS Foundation Trust

Contact SLaM

SLaM switchboard: 020 3228 6000
SLaM website: www.slam.nhs.uk

Patient Advice and Liaison Service (PALS)

PALS is here to listen and support you in whatever way we can. We want your experience at SLaM to be positive. If you are not happy about something at SLaM, we will try to help you. If you decide you want to make a complaint, we can advise you how to do this.

PALS 24hr information line: 0800 731 2864
PALS website: www.slam.nhs.uk/pals
PALS email: pals@slam.nhs.uk

Travel

For the quickest way to plan your journey to a SLaM service try Transport for London's online journey planner - www.tfl.gov.uk/journeyplanner or call 020 7222 1234 for 24hr travel information.

If you would like a large print, audio, Braille or a translated version of this leaflet then please ask us.

only

KING'S HEALTH PARTNERS

Pioneering better health for all

Reference: C4C
Date of publication: 1/3/2012
Review date: 1/3/2013

South London and Maudsley NHS
NHS Foundation Trust

Participating in Research: Information for people responsible for another adult

Biomedical Research Centre
Clinical Record Interactive Search

We are looking for people who wish to participate in research. In order to contact you about research projects for which the person you are responsible for might be suitable, we would like to ask your permission to review their health records at a future date.

This research is conducted within the Biomedical Research Centre, which is a partnership between South London and Maudsley NHS Foundation Trust (SLaM) and the Institute of Psychiatry at King's College London.

Our aim is to convert research findings into clear improvements in health for people with mental health and related problems. You can help us to do this. Volunteers who take part in research play a crucial role in improving the lives of thousands of people. We are creating a list of people who might be interested in taking part in current or future research projects.

This includes people receiving SLaM services and we are asking you as someone with appropriate responsibility whether you agree with the inclusion of the person you are responsible for on this list because it is felt that they are unable to make the decision themselves.

If you agree for this person to be part of this list, our researchers will be able to look at their SLaM records to see if they might be suitable for a research project (for example because of the symptoms they are experiencing or the medication they are taking). If they are potentially suitable for a project, a researcher will contact you and the person to discuss whether they might like to participate.

We are only asking for access to their full clinical record at SLaM. We will not need to see other records.

For peer review only

For peer review only - http://bmjopen.bmj.com/site/about/guidelines.xhtml

What type of research is carried out at the Biomedical Research Centre for Mental Health?

We carry out a large amount of research into the causes and consequences of mental health problems, from symptoms occurring in children to those occurring at the end of life. The Biomedical Research Centre particularly focuses on finding better ways to treat mental health problems and improve the lives of those affected. Our research projects have examined common mental health disorders (like anxiety and depression), dementia, psychosis, substance use disorders, and much more. The research the person might take part in could involve brain scans, puzzles, blood tests, questionnaires, interviews, computer games, or completion of a week-long diary.

For more information about our research, visit the SLaM website and search for 'BRC': http://www.slam.nhs.uk. Research is the only way to develop better treatments. Nearly all research at some point needs the help of those affected by particular health conditions in order to develop these treatments.

What will happen if I agree for this person to be on the list?

If you agree to this person being on the list, and if they do not object themselves, this simply means that you are both allowing our researchers to get in contact if they think this person might be suitable for a particular research project. It is possible that you may not be contacted at all, and it is possible that you will be contacted about a study which is not directly relevant to the symptoms they have been experiencing.

If you agree to the person's name being on this list, there is no commitment to take part in any future project. Our researcher would approach you both with information about each individual project, and would give you both time to think about participating. Both of you would then be free to agree or disagree without giving a reason for this.

All we are asking for now is your permission to review this person's SLaM health records in the future so that our researchers can make contact about research projects for which this person might be suitable.

There may not be a project suitable for this person to take part in straightaway, but we will keep people who are on the list updated about the research being done through an annual newsletter.

For peer review only

Can we choose the research projects we would like to be involved in?

Yes. Either you can specify any types of research project you will be interested in or would prefer not to be contacted about. You can let the person's care coordinator know about your preferences and these can be recorded on their electronic record.

If either of you wish to limit the number of times in a year we approach you about research studies, their care coordinator can also include this on their record, as well as any other concerns you might have.

If this person is discharged from SLaM in the future, we will ask you again about whether you wish them to remain on this list, but otherwise their name will be removed automatically. A decision to withdraw at any time, or a decision not to be put on the list in the first place, will not in any way affect the standard of care they receive.

Does this person have to be on the list?

No. It is up to both of you to decide whether you want to be on the list. This is an 'opt in' (rather than an 'opt out') system. You do not have to give a reason for not being on the list. If both of you decide to be on the list, your permission will be recorded on the person's electronic health record (i.e. on the computer system). We will give you a copy of this information for you to keep.

Either of you are free to withdraw this agreement at any time without giving a reason. If either of you want to withdraw, you can ask the person's care coordinator to alter the health record, or contact the list administrator (contact details below).

Will personal details be kept confidential?

We will comply with UK confidentiality laws to make sure that confidentiality is protected. Only our researchers who have gone through the necessary checks required by law can see personal information. However, when considering this, it is important that you realise that any piece of information recorded in the person's case record might potentially be used as a basis for making contact in the future. If you wish to participate but do not wish a particular part of their record to be used in this way then please let their care coordinator know and they can record this preference. If you are interested in finding out more about the list or participating in any of our research you can contact the list administrator by phoning 020 3228 8553 or emailing cris.administrator@kcl.ac.uk

For peer review only

South London and Maudsley NHS
NHS Foundation Trust

Contact SLaM

SLaM switchboard: 020 3228 6000
SLaM website: www.slam.nhs.uk

Patient Advice and Liaison Service (PALS)

PALS is here to listen and support you in whatever way we can. We want your experience at SLaM to be positive. If you are not happy about something at SLaM, we will try to help you. If you decide you want to make a complaint, we can advise you how to do this.

PALS 24hr information line: 0800 731 2864
PALS website: www.slam.nhs.uk/pals
PALS email: pals@slam.nhs.uk

Travel

For the quickest way to plan your journey to a SLaM service try Transport for London's online journey planner - www.tfl.gov.uk/journeyplanner or call 020 7222 1234 for 24hr travel information.

If you would like a large print, audio, Braille or a translated version of this leaflet then please ask us.

only

KING'S HEALTH PARTNERS

Reference: C4C
Date of publication: 1/3/2012
Review date: 1/3/2013

Pioneering better health for all

For peer review only - http://bmjopen.bmj.com/site/about/guidelines.xhtml

STROBE 2007 (v4) checklist of items to be included in reports of observational studies in epidemiology*

Checklist for cohort, case-control, and cross-sectional studies (combined)

Section/Topic	Item #	Recommendation	Reported on page #
**Title and abstract**	1	(*a*) Indicate the study's design with a commonly used term in the title or the abstract	2
		(*b*) Provide in the abstract an informative and balanced summary of what was done and what was found	3
**Introduction**			
Background/rationale	2	Explain the scientific background and rationale for the investigation being reported	5-7
Objectives	3	State specific objectives, including any pre-specified hypotheses	7
**Methods**			
Study design	4	Present key elements of study design early in the paper	8-9
Setting	5	Describe the setting, locations, and relevant dates, including periods of recruitment, exposure, follow-up, and data collection	8
Participants	6	(*a*) *Cohort study*—Give the eligibility criteria, and the sources and methods of selection of participants. Describe methods of follow-up<br>*Case-control study*—Give the eligibility criteria, and the sources and methods of case ascertainment and control selection. Give the rationale for the choice of cases and controls<br>*Cross-sectional study*—Give the eligibility criteria, and the sources and methods of selection of participants	9, 14
		(*b*) *Cohort study*—For matched studies, give matching criteria and number of exposed and unexposed<br>*Case-control study*—For matched studies, give matching criteria and the number of controls per case	
Variables	7	Clearly define all outcomes, exposures, predictors, potential confounders, and effect modifiers. Give diagnostic criteria, if applicable	14
Data sources/ measurement	8*	For each variable of interest, give sources of data and details of methods of assessment (measurement). Describe comparability of assessment methods if there is more than one group	14
Bias	9	Describe any efforts to address potential sources of bias	n/a
Study size	10	Explain how the study size was arrived at	n/a
Quantitative variables	11	Explain how quantitative variables were handled in the analyses. If applicable, describe which groupings were chosen and why	n/a descriptive statistics only
Statistical methods	12	(*a*) Describe all statistical methods, including those used to control for confounding	n/a descriptive statistics only
		(*b*) Describe any methods used to examine subgroups and interactions	n/a descriptive statistics only
		(*c*) Explain how missing data were addressed	n/a descriptive

For peer review only

			statistics only
		(*d*) *Cohort study*—If applicable, explain how loss to follow-up was addressed<br>*Case-control study*—If applicable, explain how matching of cases and controls was addressed<br>*Cross-sectional study*—If applicable, describe analytical methods taking account of sampling strategy	n/a descriptive statistics only
		(*e*) Describe any sensitivity analyses	n/a descriptive statistics only
**Results**			
Participants	13*	(a) Report numbers of individuals at each stage of study—eg numbers potentially eligible, examined for eligibility, confirmed eligible, included in the study, completing follow-up, and analysed	
		(b) Give reasons for non-participation at each stage	
		(c) Consider use of a flow diagram	
Descriptive data	14*	(a) Give characteristics of study participants (eg demographic, clinical, social) and information on exposures and potential confounders	14
		(b) Indicate number of participants with missing data for each variable of interest	14
		(c) *Cohort study*—Summarise follow-up time (eg, average and total amount)	
Outcome data	15*	*Cohort study*—Report numbers of outcome events or summary measures over time	
		Case-control study—Report numbers in each exposure category, or summary measures of exposure	
		Cross-sectional study—Report numbers of outcome events or summary measures	
Main results	16	(*a*) Give unadjusted estimates and, if applicable, confounder-adjusted estimates and their precision (eg, 95% confidence interval). Make clear which confounders were adjusted for and why they were included	
		(*b*) Report category boundaries when continuous variables were categorized	
		(*c*) If relevant, consider translating estimates of relative risk into absolute risk for a meaningful time period	
Other analyses	17	Report other analyses done—eg analyses of subgroups and interactions, and sensitivity analyses	n/a
**Discussion**			
Key results	18	Summarise key results with reference to study objectives	14
Limitations	19	Discuss limitations of the study, taking into account sources of potential bias or imprecision. Discuss both direction and magnitude of any potential bias	15
Interpretation	20	Give a cautious overall interpretation of results considering objectives, limitations, multiplicity of analyses, results from similar studies, and other relevant evidence	15-16
Generalisability	21	Discuss the generalisability (external validity) of the study results	15-16
**Other information**			
Funding	22	Give the source of funding and the role of the funders for the present study and, if applicable, for the original study on which the present article is based	17

For peer review only

*Give information separately for cases and controls in case-control studies and, if applicable, for exposed and unexposed groups in cohort and cross-sectional studies.

Note: An Explanation and Elaboration article discusses each checklist item and gives methodological background and published examples of transparent reporting. The STROBE checklist is best used in conjunction with this article (freely available on the Web sites of PLoS Medicine at http://www.plosmedicine.org/, Annals of Internal Medicine at http://www.annals.org/, and Epidemiology at http://www.epidem.com/). Information on the STROBE Initiative is available at www.strobe-statement.org.

For peer review only

BMJ Open

Developing a new model for patient recruitment in mental health services: a cohort study using electronic health records

Journal:	*BMJ Open*
Manuscript ID:	bmjopen-2014-005654.R1
Article Type:	Research
Date Submitted by the Author:	04-Aug-2014
Complete List of Authors:	Callard, Felicity; King's College London (Institute of Psychiatry), ; Durham University,<br>Broadbent, Matthew; South London and Maudsley NHS Fundation Trust,<br>Denis, Mike; South London and Maudsley NHS Fundation Trust,<br>Hotopf, Matthew; King's College London (Institute of Psychiatry),<br>Soncul, Murat; South London and Maudsley NHS Foundation Trust,<br>Wykes, Til; King's College London (Institute of Psychiatry),<br>Lovestone, Simon; University of Oxford, Psychiatry<br>Stewart, Robert; King's College London, Institute of Psychiatry
**Primary Subject Heading**:	Health informatics
Secondary Subject Heading:	Health informatics, Mental health, Research methods
Keywords:	Health informatics < BIOTECHNOLOGY & BIOINFORMATICS, Information management < BIOTECHNOLOGY & BIOINFORMATICS, Clinical governance < HEALTH SERVICES ADMINISTRATION & MANAGEMENT, MENTAL HEALTH, PSYCHIATRY

SCHOLARONE™
Manuscripts

For peer review only

Developing a new model for patient recruitment in mental health services: a cohort study using electronic health records

Authors:

Felicity Callard, Matthew Broadbent, Mike Denis, Matthew Hotopf, Murat Soncul, Til Wykes, Simon Lovestone, Robert Stewart

---

Dr Felicity Callard, PhD*, Durham University, Centre for Medical Humanities and Department of Geography, Lower Mountjoy, South Road, Durham DH1 3LE, UK
Reader in Social Science for Medical Humanities, Durham University

Mr Matthew Broadbent, MA, King's College London, SLaM Biomedical Research Centre Nucleus, Institute of Psychiatry, Box P092, De Crespigny Park, London SE5 8AF
Head of BRC Clinical Informatics, NIHR Biomedical Research Centre for Mental Health

Mr Mike Denis, John Eccles House, Robert Robinson Avenue
Oxford Science Park, OX4 4GP
Director of Information Strategy, Oxford Academic Health Science Centre and Network

Prof Matthew Hotopf, PhD, King's College London, Department of Psychological Medicine, Institute of Psychiatry, 10 Cutcombe Rd, London SE5 9RJ
Professor of General Hospital Psychiatry, King's College London, Institute of Psychiatry

Dr Murat Soncul, PhD, Maudsley Hospital, Denmark Hill, London SE5 8AZ
Head of Information Governance, South London and Maudsley NHS Foundation Trust

Prof Til Wykes, PhD, King's College London, Department of Psychology, Institute of Psychiatry, Box P077, De Crespigny Park, London, SE5 8AF
Professor of Clinical Psychology and Rehabilitation

Prof Simon Lovestone, PhD, University of Oxford, Department of Psychiatry, Warneford

For peer review only

Hospital, Oxford OX3 7JX

Professor of Translational Neuroscience, University of Oxford.

Prof Robert Stewart, MD, King's College London, Institute of Psychiatry, Box P063, De Crespigny Park, London SE5 8AF

Professor, King's College London, Institute of Psychiatry

* Corresponding author:

Dr Felicity Callard, Department of Geography, Lower Mountjoy, South Road, Durham, DH1 3LE, UK, felicity.callard@durham.ac.uk, +44 7736 720515

For peer review only

Abstract

Objectives: To develop a new model for patient recruitment that harnessed the full potential of electronic health records (EHRs). Gaining access to potential participants' health records to assess their eligibility for studies and allow an approach about participation ('consent to contact') is ethically, legally and technically challenging, given that medical data are usually restricted to the patient's clinical team. The research objective was to design a model for identification and recruitment to overcome some of these challenges as well as reduce the burdensome (and/or time consuming) gate-keeper role of clinicians in determining who is appropriate or not to participate in clinical research.

Setting: Large secondary mental health services context, United Kingdom.

Participants: 2,106 patients approached for 'consent for contact'. All patients in different services within the mental health trust are gradually and systematically being approached by a member of the clinical care team using the 'consent for contact' model. There are no exclusion criteria.

Primary and secondary outcome measures: Provision of 'consent for contact'.

Results: A new model (the South London and Maudsley NHS Trust Consent for Contact model (SLaM C4C)) for gaining patients' consent to contact them about research possibilities, which is built around a de-identified EHR database. The model allows researchers to contact potential participants directly. Of 2,106 patients approached by 25 October 2013, nearly three out of every four gave consent for contact (1,560 patients; 74.1%).

Conclusions: The SLaM C4C model offers an effective way of expediting recruitment into health research through using EHRs. It reduces the gatekeeper function of clinicians; gives patients greater autonomy in decisions to participate in research; and accelerates the development of a culture of active research participation. More research is needed to assess how many of those giving consent for contact subsequently consent to participate in particular research studies.

Trial registration: N/A

For peer review only

Article Summary

Strengths and limitations of the study

- A new model for gaining patients' consent to contact them about research possibilities, which is built around a robustly de-identified Electronic Health Records (EHR) database
- Currently, 3 out of every 4 patients approached are giving consent to contact using the model
- The model gives patients greater autonomy in decisions about research participation
- The model was developed through extensive patient/service user involvement and consultation
- The study does not examine whether patients who have given consent to contact go on to consent to participation in particular research studies.

For peer review only

Introduction

Recruiting patients into health research

One of the most pressing issues for researchers needing to recruit patients is how best to identify who might be suitable for their research, at the same time as adhering to legal and ethical frameworks regarding confidentiality and privacy. Medical data are, in multiple jurisdictions, generally accessible to researchers only with prior patient consent, or if the data are adequately de-identified; this means, in short, that a researcher needs to gain consent from an individual *before* accessing her personal details to adjudicate suitability for the research and before contacting her to ask about potential participation. Access to individuals' medical records is usually restricted to members of the clinical team, who thereby act as intermediaries through which contact between researchers and potential research participants is commonly made. Such a process is, however, often cumbersome and time-consuming: busy clinical teams often do not have the time to assist with recruitment; the alternative path of writing to each patient (e.g. within a general practice) to ask if she would agree to researchers accessing her medical records to assess suitability for a study is no less onerous. Furthermore, should the clinician still be a gate-holder for the patient? Is this not a remnant of medical paternalism, outdated in today's health care system and research context with citizen science and direct-to-consumer research opportunities such as the uBiome[1] project? Clinicians' desire to protect patients can, through 'gate-keeping', end up effectively denying them the opportunity to participate in research.[2] Such a problem is particularly pertinent within a mental health context. In short, it is time to allow patients greater autonomy in decisions to take part in research by reducing the gatekeeper role of clinicians – not least because such gatekeeper functions can exacerbate inequities in who is able to participate in health research.

Amidst wide concern about difficulties in recruiting to clinical studies,[3] there are calls to transform the culture of patient participation in health research (e.g. US National Institutes of Health (NIH) Road Map,[4] and England's National Health Service (NHS) Constitution).[5] The influential 2008 Data Sharing Review in the United Kingdom recommended that 'The NHS should develop a system to allow approved researchers to work with healthcare providers to identify potential patients, who may then be

For peer review only - http://bmjopen.bmj.com/site/about/guidelines.xhtml

approached to take part in clinical studies for which consent is needed'.[6] There appears, however, to be a reluctance to mandate legislatively a move away from the current model in which researchers require consent before accessing medical records.

Meanwhile, quantitative and qualitative studies in a number of countries have indicated that significant proportions of the public are cautious about giving researchers access to their medical records for recruitment purposes without prior consent, even as the public remains supportive of health research.[7-9] A mass public engagement exercise (6,000 people) in England, for example, found that only 34 per cent of adults and 10 per cent of young people agreed with extending the range of people with direct access to patient records to assist in recruitment to clinical trials.[10] Given this and the current legislative environment, it is likely that in the UK, at least, the current principles surrounding consent (i.e. that researchers are not able to access a patient's records without consent unless they are a member of the patient's clinical team) will continue to underpin research governance for some time. There is therefore a need to find ways of negotiating this governance environment that would allow more efficient identification of, and better engagement with, potential research participants, and would empower patients or service users to make their own decisions regarding which, if any, research projects to learn more about.[11-13]

Consent for contact

One response to this challenge has been the formulation of what has, in the last decade, come to be termed 'consent for contact' or 'consent to consent'. This, as the Academy of Medical Sciences puts it, comprises a 'mechanism … for individuals to give *generic consent to be contacted about suitable research opportunities*, before considering whether they consent to take part in a specific study' [italics added].[14] Notably, 'consent for contact' processes are being put in place in a range of health research facilities. A project in British Columbia, Canada has, since 2007, set up 'Permission to Contact' platforms in different outpatient health clinics (cancer, cardiac and maternal health), which have proved effective in enhancing enrollment into translational research projects.[15 16] In the United Kingdom, the UK Biobank project requires explicit consent

For peer review only - http://bmjopen.bmj.com/site/about/guidelines.xhtml

in order both to access the medical records of those joining the project, as well as potentially to re-contact these participants in the future.[17] While such generic consent is appealing for researchers, its operationalization poses a number of ethical, sociological, governance-related and technical questions (see Box 1). There is growing interest in how EHRs might be used in this regard. To date, the use of EHRs for recruitment has relied on mechanisms through which a member of the clinical team of the patient identified – via pseudonymised searching – as potentially eligible for the study is alerted and invited to contact that patient. Such use therefore currently maintains the distinction between those designing the studies and those recruiting into the studies.[18-22]

Box 1: 'Consent for contact': sociological, ethical and technical issues

- How does 'consent for contact' reshape relationships between treating clinicians, patients and researchers – in that the traditional role of clinician – as patient advocate and/or paternalistic patient protector – in relation to the researcher is downgraded in the emergence of a new kind of compact between patient and researcher?
- How does patients' giving of generic consent to be contacted affect how they subsequently respond to invitations to participate in specific research projects (i.e. do they feel more of an onus to give consent here, too, having given consent once already)?
- Does 'consent for contact' encourage an assumption that willingness to participate in health research is a moral obligation – and if so, what are the ethical, clinical and sociological implications?
- How can electronic health records be best used in developing consent for contact procedures? How should their use navigate complex questions regarding control, ownership and use of such data in relation to consent, authorization, and safe-keeping?

That many of the questions surrounding the use of EHRs for research remain unresolved, at both a conceptual and an empirical level, is demonstrated by the number of medical,

For peer review only - http://bmjopen.bmj.com/site/about/guidelines.xhtml

bioethical and governance-oriented bodies currently reflecting on them.[14 23-25] Our paper takes these discussions forward through focusing on an empirical advance: the model of consent for contact developed by the South London & Maudsley (SLaM) NHS Foundation Trust (hereafter: SLaM C4C). For whatever one's position regarding the merits or demerits of 'consent for contact', it is commonly agreed that 'further work is needed to provide guidance and models to enable appropriate access and identification of patients for research'.[26] SLaM C4C was endorsed by the UK's Information Governance Review in 2013 as an exemplar of 'an approach that allows appropriate individuals to be identified and approached to take part, without giving researchers direct access to identifiable information before consent is obtained'.[24]

In the paper we present: (i) the specifics of SLaM C4C, which we believe to be the first successful implementation of consent for contact which both harnesses the potential of de-identified electronic health records to expedite recruitment to research, and allows researchers to contact potential participants directly; (ii) descriptive statistics on how SLaM C4C is being received by patients.

Methods

Ethics committees approvals The SLaM C4C model was reviewed by the National Information Governance Board for Health and Social Care (NIGB) Ethics and Confidentiality committee (reference ECC 2–08/2010); the committee considered the model 'an elegant solution to the issue of participant recruitment' and stated that it 'strongly endorse[s] this approach to participant recruitment'. The model was approved by a Research Ethics Committee (REC) (South East London 4 REC, Ref 10/H0807/88), as well as by the SLaM Caldicott Guardian and the SLaM Trust Executive. The Clinical Record Interactive Search (CRIS), around which the model is built, was approved for use by a REC (Oxford C REC, Ref 08/H0606/71+5); its security model (which comprises both technical and procedural elements) was approved by the SLaM Trust Caldicott Guardian, and signed off by the Trust Executive.

For peer review only - http://bmjopen.bmj.com/site/about/guidelines.xhtml

The SLaM C4C model

SLaM C4C is designed to enhance patients' access to opportunities to participate in research projects of interest to them (England's NHS Constitution pledges 'to inform [patients] of research studies in which [they] may be eligible to participate').[5] It also enables researchers to identify and approach potentially eligible people. (These researchers have undergone rigorous approval procedures, which include being bound by the duties associated with a contract with King's Health Partners [Academic Health Sciences Center], which in turn involves various Human Resources checks, including a criminal record check in accordance with national Department of Health standards.) The model comprises technical and procedural elements built into the EHR case register at the NIHR Specialist Biomedical Research Centre for Mental Health at the South London & Maudsley (SLaM) NHS Foundation Trust (hereafter: SLaM BRC)). It was designed for use across SLaM, which is one of the largest mental health care providers in Europe, serving a local population of 1.2 million people, and including inpatient wards, outpatient and community services. Prior to this initiative, recruitment to clinical research in SLaM relied on the traditional system of researchers finding clinicians who were willing to identify and approach potential participants. Such nurse, medical and other clinicians may have little or no training in research and scant knowledge of any particular research programme; or they may themselves be researchers. This, together with clinical pressures and conflicting demands on time, result at best in the possibility of biased recruitment and, at worse, limit recruitment to research and obstruct patients from making decisions about participation.

The model is embedded within a search and database system (the Clinical Record Interactive Search [CRIS]), which reads and extracts information from SLaM's EHRs, removes identifiers and makes this available to researchers in de-identified format in standard analysis packages (e.g. Stata, Excel). Data consist of all clinical records on SLaM patients (unless they have requested to opt out from the register) and are searchable as both structured and free text. There are currently over 220,000 cases on the database, which increases by approximately 20,000 per year. We have described the development and characteristics of CRIS elsewhere[27]; there is also published research

For peer review only

based on analyses of CRIS data.[28] Here, we present the features of CRIS that allow research participant recruitment.

Reverse search

The case register was initially approved for use as a de-identified database whose data are searchable without consent by appropriately vetted researchers. The evaluation of this de-identification procedure demonstrated that CRIS effectively ensures patient anonymity at the same time as maximizes data (free text and structured text) that are available for research. Indeed, our bespoke pattern matching de-identification algorithm (which is applied to all structured and free text in CRIS) was shown, when evaluated, to mask patient identifiers with 98.8% precision and 97.6% recall – outperforming a comparator machine learning algorithm. (We have published a full description of the algorithm and the evaluation data.)[29] The register's technical architecture included, additionally, the potential for reverse search: allowing the identification of patients who meet given characteristics (which can be defined using structured and free text), and thereby the possibility of using CRIS to identify and approach potential participants on the basis of prior consent by individual patients or, for children or adults lacking capacity, an appropriate proxy. In effect, such a mechanism allows for the creation within the case register of a database of people ('the recruitment database') who have provided prior consent to be contacted and whose full – but de-identified – clinical records will be available to a researcher (a 'recruiter') in order to search for inclusion/exclusion criteria for specific, ethically approved studies. The researcher, once s/he has identified eligible potential participants, can then be given identifiers to access the source EHRs and approach patients about participation in that particular study (see Figure 1).

Insert Figure 1 near here

For peer review only - http://bmjopen.bmj.com/site/about/guidelines.xhtml

We have developed robust procedural mechanisms to address legal and ethical requirements and to complement this process and technical design, which we briefly outline below.

Acquiring and recording consent for contact

- The consent process for participation in the recruitment database is conducted by the patient's clinical team, most commonly by the patient's care coordinator (or, if not, by another member of the patient's care team). The process is carried out face to face. The SLaM C4C process is, additionally, being publicized across the Trust. If a SLaM patient expresses an interest in joining the C4C recruitment database by contacting a member of the SLaM C4C project team, that project worker (who is based in a clinical team and bound by the same information governance requirements as clinicians) is able to carry out the consent procedures. In addition, the patient's own care coordinator or equivalent clinician will be informed to check that there are no reasons for why that patient should not be included, as well as encouraged to fill out the research participation form within the patient's electronic health record. In other words, there is always clinician involvement in the process of consenting a patient on to the C4C recruitment database. The member of the clinical care team requesting consent receives training in the specifics of the SLaM C4C model and on how best to conduct the conversation clearly in a way that does not place undue pressure on the patient.
- Clinical staff are trained in research governance (particularly regarding consent and the assessment of mental capacity to provide consent) and the specific process of acquiring 'consent to contact'. The process, supported by an information sheet (see Appendices 1a, 1b, 1c), emphasises that patients are not being asked for consent to participate in any particular study – simply to being contacted in the future by researchers about potential participation in specified research projects based on information in their full SLaM EHR. The clinician explains, as required, more about what research is and what it might entail; what tends to be recorded in an EHR; and reminds the patient that, if she consents, she might be asked to participate in research

For peer review only

on different topics from those relating to her current treatment. A research study has been conducted to investigate how SLaM C4C consent conversations are carried out by clinicians and to ascertain which kinds of explanations of the recruitment database appear to make it more or less likely that patients will give their consent.[30]

- For patients who are children, or adults lacking capacity (where the lack of capacity is believed to be permanent), proxy assent is sought from a close friend or relative (with parental responsibility in the case of children) (see Appendices 1b and 1c). The model is attentive to the mental health context for which it was designed: where mental capacity is thought to be temporarily absent, the clinical team does not ask for consent, but aims to approach the patient at a later time in accordance with the second principle of the Mental Capacity Act.[31]
- The approach made by a clinician requesting consent to contact is tailored to the clinical context – for example, the request may be delayed for a distressed patient admitted in a crisis.
- A research participation form has been created as an additional window in the source clinical records system (see Figure 2). This includes:
  - Whether a C4C discussion has taken place with the patient (or a named proxy);
  - Whether the patient (or proxy) gave consent or not;
  - The name of the clinical staff member discussing C4C and the date discussed;
  - A free text box to record any further information. Notably, patients are encouraged to identify any particular preferences regarding research opportunities – both those that they would be particularly interested in, and kinds of research, or research topics, that they would not want to be contacted about.

For peer review only - http://bmjopen.bmj.com/site/about/guidelines.xhtml

Insert Figure 2 near here

- For patients who have consented to being contacted, the research participation form is also used to record all subsequent contacts made by researchers, and all projects the patient is participating in, has participated in, and/or has declined to participate in. If a patient withdraws consent to be contacted then the technical ability to link to his or her EHR ID will be prevented.
- The model acknowledges that patients might not be aware, at the point of giving consent to participating in the recruitment database, what information will later be contained in their medical record. All consents are revisited with the participant at the point of discharge from all SLaM services or else are deemed to have expired.

Identifying and contacting potential research participants

- In order to become a 'recruiter' – i.e. have the ability to access CRIS for approaching potential research participants – the researcher must have a substantive or honorary contract with SLaM and be an employee of one of the organisations forming King's Health Partners, the Academic Health Sciences Centre (AHSC) of which SLaM is a member. Use of CRIS for all forms of research is logged and audited and misuse would result in disciplinary action.
- Recruiters use de-identified data on CRIS to identify potential recruits. In CRIS itself, records are identified by a locally generated pseudonym (the BRC ID). This pseudonym is linked to the EHR ID number in the building of the CRIS data repository. Research users of CRIS are unable to access the link between BRC ID and EHR ID (Figure 1), which maintains the integrity of CRIS as a de-identified database.
- An Oversight Committee (which is chaired by a mental health patient) manages all access to, and monitors all use of, CRIS, and reports to the Trust Caldicott Committee (which has responsibility for ensuring the protection of patient confidentiality throughout the Trust). The committee provides operational oversight and management of CRIS – including the provision of research governance for projects

For peer review only - http://bmjopen.bmj.com/site/about/guidelines.xhtml

using CRIS; the monitoring and regular review of the effectiveness of the CRIS security model (including the de-identification processes); oversight of the administration of CRIS, including access control and maintenance, as well as the monitoring of audit logs; provision of advice on how to use CRIS; and responses to complaints related to CRIS (including from patients). There is no quorum required for individual meetings of the committee, but membership of the Committee must include patient/service user representation, a representative of the Trust's Caldicott Guardian, Trust research and development (R&D) representation and child and adolescent mental health services (CAMHS) representation. The Oversight Committee, its structure and its function are an integral component of the CRIS data resource as ethically approved.

- The Oversight Committee uses ethical, legal and scientific criteria to adjudicate such access and use. Applications to use CRIS to identify potential recruits are considered only for projects that have specific ethics and research governance approval. (In other words, projects using the SLaM C4C model must first acquire ethics and research governance approval and then approval from the Oversight Committee.) In the UK, acquiring this approval necessitates specifying how exactly patients will be recruited. This provides an additional layer of oversight, which helps to ensure that appropriate modes of approach are made to patients who have given 'consent to contact' when the topic of research is sensitive (e.g. researchers might be required to talk to the treating clinician before contacting the patient).
- The recruiter submits the pseudonymised BRC ID of potential recruits s/he wishes to contact to a trusted third party (TTP) appointed and monitored by the Oversight Committee.
- The TTP has project-specific access to the database linking the BRC IDs with the source EHR IDs. Technical specifications within the system ensure that CRIS cannot return the EHR ID of any patient who has not given consent for contact. Following reverse search, the TTP passes the EHR IDs of the potential recruits that have given consent back to the recruiter.

For peer review only - http://bmjopen.bmj.com/site/about/guidelines.xhtml

- The recruiter, through the SLaM EHR, is then able to identify and contact the patient to discuss participation in the project. Safeguards include a time limit on when the recruiter may contact patients about recruitment, and the requirement that recruiters inform each patient's care coordinator by email that they will be contacting that patient in a few days' time (to allow the opportunity for the care coordinator to suggest, for example, that an approach at the current time might not be advisable).
- The Oversight Committee monitors the research participation forms to ascertain if approaches for research participation or actual research participation appears excessive. What would constitute 'excessive' is an ethical, sociological and scientific question: what might be excessive for certain individual patients, or groups of patients, might not be for others; participating in multiple surveys might be adjudicated differently from participation in several clinical trials in a short time frame. The oversight committee is formalizing guidelines to adjudicate and respond to putatively 'excessive' research participation. Options for action include contacting the patient or clinical team about whether the patient continues to be willing to be approached about further research projects during the course of the current projects in which they are participating.

Involving and engaging stakeholders

There is increasing acknowledgement of the importance of engaging stakeholders and the public in designing ethical and technically robust procedures to guide the use of EHRs.[8 10 11 32] Mental health service users and patients made significant contributions to the development of our model. The Patient and Carer Participation Theme within the SLaM BRC took a central role in developing the model; the Oversight Committee is chaired by a mental health patient; and consultation and engagement with service user/patient groups have taken place throughout, in line with our BRC's model of involving service users/patients at all stages of translational research.[33] An annual newsletter is sent to all persons in the recruitment database summarising key findings arising from the SLaM BRC, in addition to reminding people of contact details for the database (for example, if they wish later to withdraw). The SLaM C4C model has a dedicated website, and there

For peer review only - http://bmjopen.bmj.com/site/about/guidelines.xhtml

are regular dissemination activities within the Trust to publicize the model. Details of how to contact the SLaM C4C team (who assist with requests to join the register, as well as provide information on how withdraw from the register) are also disseminated online and via other media. Those who consented to join the register but have been discharged from the Trust are regularly sent a reminder that they are on the register as well as information on how to withdraw if they wish. As of July 2014, 30 patients have withdrawn their consent from being listed on the register. (See Box 2 for a summary of key features of the SLaM C4C model.)

Box 2: Key features of the SLaM C4C model

- Extensive patient involvement throughout model's development.
- The initial contact is made by a member of the patient's clinical care team.
- Researchers gain access to identifiable information only for patients who have given prior consent.
- All recruiting researchers have undergone extensive vetting procedures, and are employed on contracts that have the same level of duties and responsibilities (and same potential penalties re disciplinary action and dismissal) as those with substantive clinical contracts.
- Patients revisit their consent to be part of the recruitment database on discharge from the service.
- Regular (at least yearly) engagement with those consenting to be part of the recruitment database – about ongoing research studies, as well as reminders about their consenting to be part of the recruitment database and details on how to withdraw consent.

For peer review only - http://bmjopen.bmj.com/site/about/guidelines.xhtml

Results

Summary statistics from the implementation of SLaM C4C

SLaM C4C started to be implemented across the South London & Maudsley NHS Trust in May 2012, and the aim is to implement C4C across the entire Trust, which serves a total of approximately 35,000 active patients, split across seven Clinical Academic Groups (CAGs). All patients are gradually being approached and asked whether they will provide 'consent for contact'. There are no exclusion criteria. Implementation commenced in services where there was already support for and/or enthusiasm about C4C. The figures from 25 October 2013 show that a total of 2,106 patients had been approached, of whom 1,560 had given consent and 546 had not: a 74.1% consent rate. Table 1 presents descriptive statistics, from October 2013, of those approached to give consent for contact, in relation to gender, ethnicity and age.

Table 1: Descriptive data regarding patients approached for SLaM C4C

	N total	N consenting	Consent rate (%)
Gender			
**Male**	1078	844	78.3
**Female**	1028	716	69.6
Self-assigned Ethnicity (amalgamated)			
**White**	1228	894	72.8
**Caribbean, African or Any other Black Background**	496	360	72.6
**Not stated**	205	168	82.0
**Other**	100	78	78.0
**Indian, Pakistani, Bangladeshi or Any other Asian Background**	77	60	77.9
Age*			
**0 to 19**	841	667	79.3
**20 to 29**	182	165	90.7
**30 to 44**	232	213	91.8
**45 to 74**	370	283	76.5
**75 +**	481	232	48.2

For peer review only - http://bmjopen.bmj.com/site/about/guidelines.xhtml

Data captured on 25 October 2013.

* NB Age distribution is not representative of the Trust as a whole, since implementation of SLaM C4C to date has significantly focused on Mental Health of Older Adults Services, and Child and Adolescent Mental Health Services

Discussion

Initial data suggest that the SLaM C4C model is capable of accelerating the development of a culture of active research participation that is founded on the ethical and effective use of electronic health records. The model has been successfully developed with significant patient/service user involvement, has received necessary approval and endorsement from all relevant governance bodies, and is currently resulting in almost three of every four patients approached agreeing to join the recruitment database. Public surveys and bioethical analyses commonly indicate that mental health is a sensitive domain in relation to EHRs, in light of concerns about potential stigma and discrimination.[32 34 35] The current consent figures in this secondary mental health services context are therefore encouraging vis-à-vis the model's transferability elsewhere.

Preliminary data from the implementation of SLaM C4C raise some interesting lines of investigation. That there are currently lower rates of consent from patients over 75 concurs with other research that indicates that older people are more likely to refuse participation in health research.[36] That there are currently higher rates of consent by men than women parallels other findings indicating that men are more likely to consent than women to a review of their medical records.[35] Variations in consent rates pose questions about possible selection bias in studies using this recruitment database. More research is needed to assess how many of those giving consent for contact subsequently consent to participate in particular research studies. Studies currently underway that are using the SLaM C4C model to recruit participants include: a longitudinal study to discover and validate biomarkers in Alzheimer's Disease; an interventional randomised, double blind exploratory study investigating the effects of an atypical antidepressant on cognition and BOLD fMRI signals in subjects remitted from depression and controls; a study examining differences in cognitive appraisals of anomalous experiences and

For peer review only - http://bmjopen.bmj.com/site/about/guidelines.xhtml

different facial emotions at a cognitive and neural level between individuals with psychotic symptoms with a need for care versus those without a need for care; and a study aiming prospectively to validate a set of questionnaires for the monitoring of treatment outcomes and side-effects (including suicidality and self-harm) in general populations and in populations known to be at elevated risk of suicide.

The model that we have presented adheres to current best practice in recruiting patients[24], most notably as regards ensuring that no identifiable patient information is available to researchers without the patient's consent. The model appears to be effective in its implementation – both in enabling the creation of a recruitment database, and in terms of acceptability to patients. We believe the model to be generalizable to other health services contexts that employ EHRs. Our intent when designing it was to guard against the erosion of trust in research – a key risk associated with the use of medical records without consent[34] – both through adhering to consent for contact principles (i.e. ensuring that patients are explicitly asked for their consent before their medical records can be looked at for research purposes) and committing regularly to engage with members of the recruitment database about ongoing research and about their current willingness to be contacted about potential research possibilities. We designed the model to allow patients greater autonomy in decisions to take part in research, through lessening the gatekeeper role of clinicians: evidence from a prospective cohort study and a qualitative process evaluation indicates that such gatekeeper functions can impede equitable access to research.[13 37]

A new culture of research participation?

The use, linkage and further development of large EHR datasets are likely to transform relations between researchers, clinicians, patients and their data. The implementation of SLaM C4C, across a large mental health provider, generates, we believe, effectively a field site or laboratory in which to study these potential transformations. The Oversight Committee regularly captures descriptive data that give broad indications of how implementation of SLaM C4C is proceeding (both as regards patients approached, and as

For peer review only - http://bmjopen.bmj.com/site/about/guidelines.xhtml

regards the number and type of research studies applying to use SLaM C4C). In addition, SLaM BRC is planning additional research and evaluation studies (see Box 3 for indicative research studies that address important questions associated with the use of EHRs for health research)[32 35].

Box 3: Research and evaluation questions raised by the SLaM C4C model

- *Conveying consent for contact to patients.* One current study is using focus groups and reiterative methods to ask: What kinds of explanations of consent for contact are most clearly comprehensible to patients?
- *Patients' experience of consent for contact.* How do patients interpret the process of giving (or refusing) generic consent to access medical records? Does the SLaM C4C model affect their sense of autonomy vis-à-vis decisions about participating in health research? How often do those who give consent for contact go on to participate in research?
- *Researchers' experience consent for contact.* Does it facilitate recruitment (in terms of ease of recruiting, time to reach recruitment targets)? Does the possibility of being able to use free-text in CRIS to identify and cluster patients change how studies are designed?
- *Clinicians' experience of consent for contact (both those who make the approach for 'consent for contact' and others).* How, practically, do clinicians carry out and record consent for contact discussions with their patients? Does consent for contact alter relationships between clinicians and patients?
- *Differential rates of those consenting.* How and why do rates of consent vary across demographic groups and types of service? Does this produce selection bias in studies recruiting from the database?
- *Consent for contact as a potential mechanism to help facilitate public engagement vis-à-vis participation in health research and broader use of, and trust in, EHRs.*

For peer review only - http://bmjopen.bmj.com/site/about/guidelines.xhtml

Our BRC's ongoing collection of survey and audit data, as well as planned research studies, comprise a powerful means through which to interrogate and analyse the sociological, ethical, technical and governance-related ramifications of large-scale EHR implementation of consent for contact, in which clinicians no longer provide the primary conduit for patient participation in research.

Contributorship Statement

The idea for the manuscript was conceived by FC in conversation with RS and SL. FC drafted the manuscript, with substantial text contributions from MB, SL and RS. All authors were involved in critical revision of the manuscript before submission, and approved the final manuscript. All authors developed and/or refined the consent for contact model described in the paper. FC is the guarantor. FC is Chair of the CRIS Oversight Committee and was Chair of the Security and Confidentiality Procedures Working Group that developed the security model for CRIS; MB project managed the development of CRIS; MD directed information strategy within SLaM during the development of CRIS and SLaM C4C; MH developed the protocol for patients lacking mental capacity; MS is Head of Information Governance and the Caldicott representative at SLaM; TW is Patient and Carer Participation Theme Lead for the Biomedical Research Centre for Mental Health; SL was director of the Biomedical Research Centre for Mental Health during the development of CRIS and SLaM C4C; RS led the academic development of CRIS. RS, SL and MH secured funding for the work.

Competing Interests

SL is co-ordinator of the European Medical Information Framework (www.emif.eu), a public private partnership with funding from EU and pharmaceutical companies that seeks to make use of both research and routine clinical data for research.
There are no other competing interests.

For peer review only - http://bmjopen.bmj.com/site/about/guidelines.xhtml

Acknowledgements

We thank the NIGB Ethics and Confidentiality Committee for its helpful comments on and input into the final design of the model for recruitment. We also thank the CRIS Oversight Committee and the CRIS Security & Confidentiality Procedures Working Group. Andrea Fernandes (CRIS Administrator), Jenny Liebscher (Research & Development [R&D] Governance and Delivery Manager for the SLaM and Institute of Psychiatry R&D Office), Sheri Oduola (Consent for Contact (C4C) Project Manager) and Megan Pritchard (CRIS Training and Development Lead) provided important details on how the SLaM C4C model is being implemented in practice.

Funding The authors acknowledge financial support from the National Institute for Health Research (NIHR) Specialist Biomedical Research Centre for Mental Health award to the South London and Maudsley NHS Foundation Trust and the Institute of Psychiatry, King's College London. The development of the SLAM BRC Case Register was funded by a Capital Award from the UK NIHR and is further supported through the BRC Nucleus funded jointly by the Guy's and St Thomas' Trustees and South London and Maudsley Special Trustees.

Authors FC, MB, MH, TW, SL and RS – as well as CRIS itself – are or have been part-funded by the National Institute for Health Research (NIHR) Biomedical Research Centre and Dementia Biomedical Research Unit at South London and Maudsley NHS Foundation Trust and King's College London for this research. SL and TW acknowledge the NIHR for their Senior Investigator Awards. The development of the SLAM BRC Case Register was funded by a Capital Award from the UK NIHR and is further supported through the BRC Nucleus funded jointly by the Guy's and St Thomas' Trustees and South London and Maudsley Special Trustees. The funders had no role in study design, data collection and analysis, decision to publish, or preparation of the manuscript.

For peer review only - http://bmjopen.bmj.com/site/about/guidelines.xhtml

Contributorship Statement:

The idea for the manuscript was conceived by FC in conversation with RS and SL. FC drafted the manuscript, with substantial text contributions from MB, SL and RS. All authors were involved in critical revision of the manuscript before submission, and approved the final manuscript. All authors developed and/or refined the consent for contact model described in the paper. FC is the guarantor. FC is Chair of the CRIS Oversight Committee and was Chair of the Security and Confidentiality Procedures Working Group that developed the security model for CRIS; MB project managed the development of CRIS; MD directed information strategy within SLaM during the development of CRIS and SLaM C4C; MH developed the protocol for patients lacking mental capacity; MS is Head of Information Governance and the Caldicott representative at SLaM; TW is Patient and Carer Participation Theme Lead for the Biomedical Research Centre for Mental Health; SL was director of the Biomedical Research Centre for Mental Health during the development of CRIS and SLaM C4C; RS led the academic development of CRIS. RS, SL and MH secured funding for the work.

Competing Interests:

Simon Lovestone is co-ordinator of the European Medical Information Framework (www.emif.eu), a public private partnership with funding from EU and pharmaceutical companies that seeks to make use of both research and routine clinical data for research. There are no other conflicts of interest.

Data Sharing

No additional data available.

For peer review only

References

1. uBiome. Secondary. http://ubiome.com.
2. Higginson IJ, Evans CJ, Grande G, et al. Evaluating complex interventions in end of life care: the MORECare statement on good practice generated by a synthesis of transparent expert consultations and systematic reviews. BMC Med 2013;**11**:111 doi: 10.1186/1741-7015-11-111[published Online First: Epub Date]|.
3. Ross S, Grant A, Counsell C, et al. Barriers to participation in randomised controlled trials: a systematic review. J Clin Epidemiol 1999;**52**(12):1143-56
4. Zerhouni EA. Translational research: moving discovery to practice. Clin Pharmacol Ther 2007;**81**(1):126-8 doi: 10.1038/sj.clpt.6100029[published Online First: Epub Date]|.
5. Department of Health. The NHS Constitution for England: Department of Health, 2013.
6. Thomas R, Walport M. Data Sharing Review Report. London: Ministry of Justice, 2008.
7. Buckley BS, Murphy AW, MacFarlane AE. Public attitudes to the use in research of personal health information from general practitioners' records: a survey of the Irish general public. J Med Ethics 2011;**37**(1):50-5 doi: 10.1136/jme.2010.037903[published Online First: Epub Date]|.
8. Armstrong V, Barnett J, Cooper H, et al. Public perspectives on the governance of biomedical research: a qualitative study in a deliberative context. London: Wellcome Trust, 2007.
9. Willison DJ, Schwartz L, Abelson J, et al. Alternatives to project-specific consent for access to personal information for health research: what is the opinion of the Canadian public? J Am Med Inform Assoc 2007;**14**(6):706-12 doi: 10.1197/jamia.M2457[published Online First: Epub Date]|.
10. new economics foundation. Who sees what: exploring public views on personal electronic health records. London: new economics foundation, 2010.
11. Academy of Medical Sciences. Personal data for public good: using health information in medical research. London: The Academy of Medical Sciences, 2006.
12. Hewison J, Haines A. Overcoming barriers to recruitment in health research. BMJ 2006;**333**(7562):300-2 doi: 10.1136/bmj.333.7562.300[published Online First: Epub Date]|.
13. Knowles RL, Bull C, Wren C, et al. Ethics, governance and consent in the UK: implications for research into the longer-term outcomes of congenital heart defects. Arch Dis Child 2011;**96**(1):14-20 doi: 10.1136/adc.2008.152975[published Online First: Epub Date]|.
14. Academy of Medical Sciences. A new pathway for the regulation and governance of health research. London: The Academy of Medical Sciences, 2011.
15. Cheah S, O'Donoghue S, Daudt H, et al. Permission to contact (PTC)--a strategy to enhance patient engagement in translational research. Biopreserv Biobank

For peer review only - http://bmjopen.bmj.com/site/about/guidelines.xhtml

2013;**11**(4):245-52 doi: 10.1089/bio.2013.0023[published Online First: Epub Date]|.
16. LeBlanc J, Dee S, Braun L, et al. Impact of a Permission to Contact (PTC) platform on biobank enrollment and efficiency. Biopreserv Biobank 2013;**11**(3):144-8 doi: 10.1089/bio.2013.0004[published Online First: Epub Date]|.
17. UK Biobank. Biobank Information Leaflet, 2010.
18. Brooks CJ, Stephens JW, Price DE, et al. Use of a patient linked data warehouse to facilitate diabetes trial recruitment from primary care. Prim Care Diabetes 2009;**3**(4):245-8 doi: 10.1016/j.pcd.2009.06.004[published Online First: Epub Date]|.
19. Embi PJ, Jain A, Clark J, et al. Effect of a clinical trial alert system on physician participation in trial recruitment. Arch Intern Med 2005;**165**(19):2272-7 doi: 10.1001/archinte.165.19.2272[published Online First: Epub Date]|.
20. McGregor J, Brooks C, Chalasani P, et al. The Health Informatics Trial Enhancement Project (HITE): Using routinely collected primary care data to identify potential participants for a depression trial. Trials 2010;**11**:39 doi: 10.1186/1745-6215-11-39[published Online First: Epub Date]|.
21. Ainsworth J, Buchan I. Preserving consent-for-consent with feasibility-assessment and recruitment in clinical studies: FARSITE architecture. Stud Health Technol Inform 2009;**147**:137-48
22. Stuardi T, Cox H, Torgerson DJ. Database recruitment: a solution to poor recruitment in randomized trials? Fam Pract 2011;**28**(3):329-33 doi: 10.1093/fampra/cmq108[published Online First: Epub Date]|.
23. Nuffield Council on Bioethics. New Working Party on Biodata. Secondary New Working Party on Biodata 2013. http://www.nuffieldbioethics.org/news/new-working-party-biodata.
24. Caldicott F. Information: to share or not to share?: the information governance review. London: Department of Health, 2013.
25. Great Britain. Department of H. Information: to share or not to share?: Government response to the Caldicott Review. In: Great Britain. Department of H, ed.: Department of Health, 2013.
26. Health Research Authority. The HRA welcomes the publication of Dame Fiona Caldicott's Information Governance Review. 2013
27. Stewart R, Soremekun M, Perera G, et al. The South London and Maudsley NHS Foundation Trust Biomedical Research Centre (SLAM BRC) case register: development and descriptive data. BMC Psychiatry 2009;**9**:51 doi: 10.1186/1471-244X-9-51[published Online First: Epub Date]|.
28. Chang CK, Hayes RD, Broadbent M, et al. All-cause mortality among people with serious mental illness (SMI), substance use disorders, and depressive disorders in southeast London: a cohort study. BMC Psychiatry 2010;**10**:77 doi: 10.1186/1471-244X-10-77[published Online First: Epub Date]|.
29. Fernandes AC, Cloete D, Broadbent MT, et al. Development and evaluation of a de-identification procedure for a case register sourced from mental health electronic records. BMC Med Inform Decis Mak 2013;**13**:71 doi: 10.1186/1472-6947-13-71[published Online First: Epub Date]|.

For peer review only - http://bmjopen.bmj.com/site/about/guidelines.xhtml

30. Robotham D, Riches S, Perdue I, et al. Consenting for contact? Linking electronic health records to a research register within psychosis services. Under review
31. *Mental Capacity Act 2005 : code of practice*. London: TSO, 2007.
32. Ipsos MORI. The use of personal health information in medical research: general public consultation. Final report. London: Medical Research Council, 2007.
33. Callard F, Rose D, Wykes T. Close to the bench as well as at the bedside: involving service users in all phases of translational research. Health Expect 2012;**15**(4):389-400 doi: 10.1111/j.1369-7625.2011.00681.x[published Online First: Epub Date]|.
34. Rothstein MA. Is deidentification sufficient to protect health privacy in research? Am J Bioeth 2010;**10**(9):3-11 doi: 10.1080/15265161.2010.494215[published Online First: Epub Date]|.
35. Hill EM, Turner EL, Martin RM, et al. "Let's get the best quality research we can": public awareness and acceptance of consent to use existing data in health research: a systematic review and qualitative study. BMC Med Res Methodol 2013;**13**:72 doi: 10.1186/1471-2288-13-72[published Online First: Epub Date]|.
36. Williams B, Irvine L, McGinnis AR, et al. When "no" might not quite mean "no"; the importance of informed and meaningful non-consent: results from a survey of individuals refusing participation in a health-related research project. BMC Health Serv Res 2007;**7**:59 doi: 10.1186/1472-6963-7-59[published Online First: Epub Date]|.
37. Patterson S, Kramo K, Soteriou T, et al. The great divide: a qualitative investigation of factors influencing researcher access to potential randomised controlled trial participants in mental health settings. J Ment Health 2010;**19**(6):532-41 doi: 10.3109/09638237.2010.520367[published Online First: Epub Date]|.

FIGURE LEGENDS:

Figure 1: The SLaM ‘consent for contact’ model

Figure 2: Screen shot of the SLaM C4C Patient Participation Form in the original source EHR (Part I)

Figure 2: Screen shot of the SLaM C4C Patient Participation Form in the original source EHR (part II)

For peer review only

Realising the potential for clinical research using electronic health records and 'consent for contact' in a mental health context: development and descriptive data

Authors:

Felicity Callard, Matthew Broadbent, Mike Denis, Matthew Hotopf, Murat Soncul, Til Wykes, Simon Lovestone, Robert Stewart

---

Dr Felicity Callard, PhD*, ~~King's College London, Service User Research Enterprise, Box P034, Institute of Psychiatry, De Crespigny Park, London SE5 8AF~~

~~Visiting Researcher, King's College London, Institute of Psychiatry~~Durham University, Centre for Medical Humanities and Department of Geography, Lower Mountjoy, South Road, Durham DH1 3LE, UK

Reader in Social Science for Medical Humanities, Durham University

Mr Matthew Broadbent, MA, King's College London, SLaM Biomedical Research Centre Nucleus, Institute of Psychiatry, Box P092, De Crespigny Park, London SE5 8AF

Head of BRC Clinical Informatics, NIHR Biomedical Research Centre for Mental Health

Mr Mike Denis, John Eccles House, Robert Robinson Avenue

Oxford Science Park, OX4 4GP

~~Leonard House, 7 Newman Road, Bromley, BR1 1RJ~~

Director of Information Strategy, Oxford Academic Health Science Centre and Network

~~Director of Information Strategy, South London and Maudsley NHS Foundation Trust~~

Prof Matthew Hotopf, PhD, King's College London, Department of Psychological Medicine, Institute of Psychiatry, 10 Cutcombe Rd, London SE5 9RJ

Professor of General Hospital Psychiatry, King's College London, Institute of Psychiatry

Dr Murat Soncul, PhD, Maudsley Hospital, Denmark Hill, London SE5 8AZ

Formatted: Right: 0"

Formatted: Font: Times, 12 pt

Formatted: Font: Times, 12 pt

Formatted: Line spacing: 1.5 lines

Formatted: Font: (Default) Times, 12 pt, Font color: Auto

Formatted: Font: (Default) Times, 12 pt

Formatted: Font: (Default) Times, 12 pt

For peer review only

For peer review only - http://bmjopen.bmj.com/site/about/guidelines.xhtml

Head of Information Governance, South London and Maudsley NHS Foundation Trust

Prof Til Wykes, PhD, King's College London, Department of Psychology, Institute of Psychiatry, Box P077, De Crespigny Park, London, SE5 8AF

Professor of Clinical Psychology and Rehabilitation

Prof Simon Lovestone, PhD, University of Oxford, Department of Psychiatry, Warneford Hospital, Oxford OX3 7JX

Professor of Translational Neuroscience, University of Oxford.~~King's College London, Institute of Psychiatry, Box P070, De Crespigny Park, London SE5 8AF~~

~~Professor of Old Age Psychiatry, King's College London, Institute of Psychiatry~~

Prof Robert Stewart, MD, King's College London, Institute of Psychiatry, Box P063, De Crespigny Park, London SE5 8AF

Professor, King's College London, Institute of Psychiatry

* Corresponding author:

Dr Felicity Callard, Department of Geography, Lower Mountjoy, South Road, Durham, DH1 3LE, UK~~Box P034, Institute of Psychiatry, De Crespigny Park, London SE5 8AF~~, ~~felicity.callard@kcl.ac.uk~~felicity.callard@durham.ac.uk, +44 7736 720515

Formatted: Line spacing: 1.5 lines

Formatted: Font: 12 pt

Formatted: Font: (Default) Times, 12 pt

Formatted: Font: 12 pt

Formatted: Right: 0"

Formatted: Font: Times, 12 pt

Formatted: Font: Times, 12 pt

Formatted: Default Paragraph Font, Font: (Default) Cambria

For peer review only

For peer review only - http://bmjopen.bmj.com/site/about/guidelines.xhtml

Abstract

Objectives: ~~This research aimed to~~To develop a new model for patient recruitment that harnessed the full potential of electronic health records (EHRs). Gaining access to potential participants' health records to assess their eligibility for studies and allow an approach about participation ('consent to contact') is ethically, legally and technically challenging, given that medical data are usually restricted to the patient's clinical team. The research objective was to design ~~to~~ a model for identification and recruitment to overcome some of these challenges as well as reduce the burdensome (and/or time consuming) gate-keeper ~~function~~ role of clinicians in determining who is appropriate or not to participate in clinical research.

Setting: Large secondary mental health services context, United Kingdom.

Participants: 2,106 patients approached for 'consent for contact'. All patients in different services within the mental health trust are gradually and systematically being approached by a member of the clinical care team using the 'consent for contact' model. There are no exclusion criteria.

Primary and secondary outcome measures: Provision of 'consent for contact'.

Results: A new model (the South London and Maudsley NHS Trust Consent for Contact model (SLaM C4C)) for gaining patients' consent to contact them about research possibilities, which is built around a de-identified EHR database. The model allows researchers to contact potential participants directly. Of ~~the~~ 2,106 patients approached by 25 October 2013, nearly three out of every four gave consent for contact (1,560 patients; 74.1%).

Conclusions: The SLaM C4C model offers an effective way of expediting recruitment into health research through ~~the use of~~using EHRs. It reduces the gatekeeper function of clinicians; gives patients greater autonomy in decisions to ~~take part~~participate in research; and accelerates the development of a culture of active research participation. More research is needed to assess how many of those giving consent for contact subsequently consent to participate in particular research studies.

Trial registration: N/A

~~Extra data are available by emailing felicity.callard@kcl.ac.uk~~

Funding The authors acknowledge financial support from the National Institute for Health Research (NIHR) Specialist Biomedical Research Centre for Mental Health award to the South London and Maudsley NHS Foundation Trust and the Institute of Psychiatry,

For peer review only - http://bmjopen.bmj.com/site/about/guidelines.xhtml

King's College London. The development of the SLAM BRC Case Register was funded by a Capital Award from the UK NIHR and is further supported through the BRC Nucleus funded jointly by the Guy's and St Thomas' Trustees and South London and Maudsley Special Trustees.

Article Summary

Strengths and limitations of the study

- A new model for gaining patients' consent to contact them about research possibilities, which is built around a robustly de-identified Electronic Health Records (EHR) database
- Currently, 3 out of every 4 patients approached are giving consent to contact using the model
- The model gives patients greater autonomy in decisions about research participation
- The model was developed through extensive patient/service user involvement and consultation
- The study does not examine whether patients who have given consent to contact go on to consent to participation in particular research studies.

For peer review only - http://bmjopen.bmj.com/site/about/guidelines.xhtml

Introduction

Recruiting patients into health research

One of the most pressing issues for researchers needing to recruit patients is how best to identify who might be suitable for their research, at the same time as adhering to legal and ethical frameworks regarding confidentiality and privacy. Medical data are, in multiple jurisdictions, generally accessible to researchers only with prior patient consent, or if the data are adequately de-identified; this means, in short, that a researcher needs to gain consent from an individual *before* accessing her personal details to adjudicate suitability for the research and before contacting her to ask about potential participation. Access to individuals' medical records is usually restricted to members of the clinical team, who thereby act as intermediaries through which contact between researchers and potential research participants is commonly made. Such a process is, however, often cumbersome and time-consuming: busy clinical teams often do not have the time to assist with recruitment; the alternative path of writing to each patient (e.g. within a general practice) to ask if she would agree to researchers accessing her medical records to assess suitability for a study is no less onerous. Furthermore, should the clinician still be a gate-holder for the patient? Is this not a remnant of medical paternalism, outdated in today's health care system and research context with citizen science and direct-to-consumer research opportunities such as the uBiome[1] project? Clinicians' desire to protect patients can, through 'gate-keeping', end up effectively denying them the opportunity to participate in research.[2] Such a problem is particularly pertinent within a mental health context. In short, it is time to allow patients greater autonomy in decisions to take part in research by reducing the gatekeeper role of clinicians – not least because such gatekeeper functions can exacerbate inequities in who is able to participate in health research.

Amidst wide concern about difficulties in recruiting to clinical studies,[3] there are calls to transform the culture of patient participation in health research (e.g. US National Institutes of Health (NIH) Road Map,[4] and England's National Health Service (NHS) Constitution).[5] The influential 2008 Data Sharing Review in the United Kingdom

For peer review only - http://bmjopen.bmj.com/site/about/guidelines.xhtml

recommended that ‘The NHS should develop a system to allow approved researchers to work with healthcare providers to identify potential patients, who may then be approached to take part in clinical studies for which consent is needed’.[6] There appears, however, to be a reluctance to mandate legislatively a move away from the current model in which researchers require consent before accessing medical records.

Meanwhile, quantitative and qualitative studies in a number of countries have indicated that significant proportions of the public are cautious about giving researchers access to their medical records for recruitment purposes without prior consent, even as the public remains supportive of health research.[7-9] A ~~recent~~ mass public engagement exercise (6,000 people) in England, for example, found that only 34 per cent of adults and 10 per cent of young people agreed with extending the range of people with direct access to patient records to assist in recruitment to clinical trials.[10] Given this and the current legislative environment, it is likely that in the UK, at least, the current principles surrounding consent (i.e. that researchers are not able to access a patient’s records without consent unless they are a member of the patient’s clinical team) will continue to underpin research governance for some time. There is therefore a~~n urgent scientific~~ need to find ways of negotiating this governance environment that would allow more efficient identification of, and better engagement with, potential research participants, and would empower patients or service users to make their own decisions regarding which, if any, research projects to learn more about.[11-13]

Consent for contact

One response to this challenge has been the formulation of what has, in the last decade, come to be termed ‘consent for contact’ or ‘consent to consent’. This, as the Academy of Medical Sciences puts it, comprises a ‘mechanism … for individuals to give *generic consent to be contacted about suitable research opportunities*, before considering whether they consent to take part in a specific study’ [italics added].[14] Notably, ‘consent for contact’ processes are being put in place in a range of health research facilities. A project in British Columbia, Canada has, since 2007, set up ‘Permission to Contact’ platforms in different outpatient health clinics (cancer, cardiac and maternal

Formatted: Font: Times, 12 pt

For peer review only - http://bmjopen.bmj.com/site/about/guidelines.xhtml

health), which have proved effective in enhancing enrollment into translational research projects.[15 16] In the United Kingdom, the UK Biobank project requires explicit consent in order both to access the medical records of those joining the project, as well as potentially to re-contact these participants in the future.[17] While such generic consent is appealing for researchers, its operationalization poses a number of ethical, sociological, governance-related and technical questions (see Figure 1). There is growing interest in how EHRs might be used in this regard. To date, the use of EHRs for recruitment has relied on mechanisms through which a member of the clinical team of the patient identified – via pseudonymised searching – as potentially eligible for the study is alerted and invited to contact that patient. Such use therefore currently maintains the distinction between those designing the studies and those recruiting into the studies.[18-22]

Insert Figure 1 near here

That many of the questions surrounding the use of EHRs for research remain unresolved, at both a conceptual and an empirical level, is demonstrated by the number of medical, bioethical and governance-oriented bodies currently reflecting on them.[14 23-25] Our paper takes these discussions forward through focusing on an empirical advance: the model of consent for contact developed by the South London & Maudsley (SLaM) NHS Foundation Trust (hereafter: SLaM C4C). For whatever one's position regarding the merits or demerits of 'consent for contact', it is commonly agreed that 'further work is needed to provide guidance and models to enable appropriate access and identification of patients for research'.[26] SLaM C4C was endorsed by the UK's Information Governance Review in 2013 as an exemplar of 'an approach that allows appropriate individuals to be identified and approached to take part, without giving researchers direct access to identifiable information before consent is obtained'.[24]

In the paper we present: (i) the specifics of SLaM C4C, which we believe to be the first successful implementation of consent for contact which both harnesses the potential of de-identified electronic health records to expedite recruitment to research, and allows

For peer review only - http://bmjopen.bmj.com/site/about/guidelines.xhtml

researchers to contact potential participants directly; (ii) descriptive statistics on how SLaM C4C is being received by patients.

Methods

Ethics committees approvals The SLaM C4C model was reviewed by the National Information Governance Board for Health and Social Care (NIGB) Ethics and Confidentiality committee (reference ECC 2–08/2010); the committee considered the model 'an elegant solution to the issue of participant recruitment' and stated that it 'strongly endorse[s] this approach to participant recruitment'. The model was approved by a Research Ethics Committee (REC) (South East London 4 REC, Ref 10/H0807/88), as well as by the SLaM Caldicott Guardian and the SLaM Trust Executive. The Clinical Record Interactive Search (CRIS), around which the model is built, was approved for use by a ~~Research Ethics Committee~~REC (Oxford C REC, Ref 08/H0606/71+5); its security model (which comprises both technical and procedural elements) was approved by the SLaM Trust Caldicott Guardian, and signed off by the Trust Executive. ~~Full details of all approvals are available as required.~~

Formatted: Font: (Default) Times New Roman, Not Italic

Formatted: Font: (Default) Times New Roman, Not Italic

Formatted: Font: (Default) Times New Roman, Not Bold

The SLaM C4C model

SLaM C4C is designed to enhance patients' access to opportunities to participate in research projects of interest to them (England's NHS Constitution pledges 'to inform [patients] of research studies in which [they] may be eligible to participate').[5] It also enables researchers ~~(who have undergone rigorous approval procedures)~~ to identify and approach potentially eligible people. (These researchers have undergone rigorous approval procedures, which include being bound by the duties associated with a contract with King's Health Partners [Academic Health Sciences Center], which in turn involves various Human Resources checks, including a criminal record check in accordance with national Department of Health standards.) The model comprises technical and procedural elements built into the EHR case register at the NIHR Specialist Biomedical Research Centre for Mental Health at the South London & Maudsley (SLaM) NHS Foundation Trust (hereafter: SLaM BRC)). It was designed for use across SLaM, which is one of the

For peer review only - http://bmjopen.bmj.com/site/about/guidelines.xhtml

largest mental health care providers in Europe, serving a local population of 1.2 million people, and including inpatient wards, outpatient and community services. Prior to this initiative, recruitment to clinical research in SLaM relied on the traditional system of researchers finding clinicians who were willing to identify and approach potential participants. Such nurse, medical and other clinicians may have little or no training in research and scant knowledge of any particular research programme; or they may themselves be researchers. This, together with clinical pressures and conflicting demands on time, result at best in the possibility of biased recruitment and, at worse, limit recruitment to research and obstruct patients from making decisions about participation.

The model is embedded within a search and database system (the Clinical Record Interactive Search [CRIS]), which reads and extracts information from SLaM's EHRs, removes identifiers and makes this available to researchers in de-identified format in standard analysis packages (e.g. Stata, Excel). Data consist of all clinical records on SLaM patients (unless they have requested to opt out from the register) and are searchable as both structured and free text. There are currently over 220,000 cases on the database, which increases by approximately 20,000 per year. We have described the development and characteristics of CRIS elsewhere[27]; there is also published research based on analyses of CRIS data.[28] Here, we present the features of CRIS that allow research participant recruitment.

Reverse search

The case register was initially approved for use as a de-identified database whose data are searchable without consent by appropriately vetted researchers. The evaluation of this de-identification procedure demonstrated that CRIS effectively ensures patient anonymity at the same time as maximizes data (free text and structured text) that are available for research. Indeed, our bespoke pattern matching de-identification algorithm (which is applied to all structured and free text in CRIS) was shown, when evaluated, to mask patient identifiers with 98.8% precision and 97.6% recall – outperforming a comparator machine learning algorithm. (We have published a full description of the algorithm and the evaluation data.)[29] The register's technical architecture included, additionally, the

For peer review only - http://bmjopen.bmj.com/site/about/guidelines.xhtml

potential for reverse search: allowing the identification of patients who meet given characteristics (which can be defined using structured and free text), and thereby the possibility of using CRIS to identify and approach potential participants on the basis of prior consent by individual patients or, for children or adults lacking capacity, an appropriate proxy. In effect, such a mechanism allows for the creation within the case register of a database of people (‘the recruitment database’) who have provided prior consent to be contacted and whose full – but de-identified – clinical records will be available to a researcher (a ‘recruiter’) in order to search for inclusion/exclusion criteria for specific, ethically approved studies. The researcher, once s/he has identified eligible potential participants, can then be given identifiers to access the source EHRs and approach patients about participation in that particular study (see Figure 2).

Insert Figure 2 near here

We have developed robust procedural mechanisms to address legal and ethical requirements and to complement this process and technical design, which we briefly outline below.

Acquiring and recording consent for contact

- The consent process for participation in the recruitment database is conducted ~~entirely~~ by the patient’s clinical team, most commonly by the patient’s care coordinator (or, if not, by another member of the patient’s care team). The process is carried out face to face. The SLaM C4C process is, additionally, being publicized across the Trust. If a SLaM patient expresses an interest in joining the C4C recruitment database by contacting a member of the SLaM C4C project team, that project worker (who is based in a clinical team and bound by the same information governance requirements as clinicians) is able to carry out the consent procedures. In addition, the patient’s own care coordinator or equivalent clinician will be informed to check that there are no reasons for why that patient should not be included, as well as encouraged to fill out the research participation form within the patient’s electronic health record. In other words, there is always clinician involvement in the process of consenting a

Formatted: Justified, Indent: Left: 0", Right: -0", Space After: 6 pt, Line spacing: 1.5 lines, Bulleted + Level: 1 + Aligned at: 0.25" + Indent at: 0.5", Widow/Orphan control, Adjust space between Latin and Asian text, Adjust space between Asian text and numbers

For peer review only - http://bmjopen.bmj.com/site/about/guidelines.xhtml

For peer review only

patient on to the C4C recruitment database. The member of the clinical care team requesting consent receives training in the specifics of the SLaM C4C model and on how best to conduct the conversation clearly in a way that does not place undue pressure on the patient. [30].

Formatted: Font: Times, 12 pt

- Clinical staff are trained in research governance (particularly regarding consent and the assessment of mental capacity to provide consent) and the specific process of acquiring 'consent to contact'. The process, supported by an information sheet (see Appendices 1a–c), emphasises that patients are not being asked for consent to participate in any particular study – simply to being contacted in the future by researchers about potential participation in specified research projects based on information in their full SLaM EHR. The clinician explains, as required, more about what research is and what it might entail; what tends to be recorded in an EHR; and reminds the patient that, if she consents, she might be asked to participate in research on different topics from those relating to her current treatment. A research study has been conducted to investigate how SLaM C4C consent conversations are carried out by clinicians and to ascertain which kinds of explanations of the recruitment database appear to make it more or less likely that patients will give their consent.[30]
- For patients who are children, or adults lacking capacity (where the lack of capacity is believed to be permanent), proxy assent is sought from a close friend or relative (with parental responsibility in the case of children) (see Appendices 1b and 1c). The model is attentive to the mental health context for which it was designed: where mental capacity is thought to be temporarily absent, the clinical team does not ask for consent, but aims to approach the patient at a later time in accordance with the second principle of the Mental Capacity Act.[31]
- The approach made by a clinician requesting consent to contact is tailored to the clinical context – for example, the request may be delayed for a distressed patient admitted in a crisis.
- A research participation form has been created as an additional window in the source clinical records system (see Figure 3). This includes:

For peer review only - http://bmjopen.bmj.com/site/about/guidelines.xhtml

- Whether a C4C discussion has taken place with the patient (or a named proxy);
- Whether the patient (or proxy) gave consent or not;
- The name of the clinical staff member discussing C4C and the date discussed;
- A free text box to record any further information. Notably, patients are encouraged to identify any particular preferences regarding research opportunities – both those that they would be particularly interested in, and kinds of research, or research topics, that they would not want to be contacted about.

Insert Figure 3 near here

- For patients who have consented to being contacted, the research participation form is also used to record all subsequent contacts made by researchers, and all projects the patient is participating in, has participated in, and/or has declined to participate in. If a patient withdraws consent to be contacted then the technical ability to link to his or her EHR ID will be prevented.
- The model acknowledges that patients might not be aware, at the point of giving consent to participating in the recruitment database, what information will later be contained in their medical record. All consents are revisited with the participant at the point of discharge from all SLaM services or else are deemed to have expired.

Identifying and contacting potential research participants

- In order to become a 'recruiter' – i.e. have the ability to access CRIS for approaching potential research participants – the researcher must have a substantive or honorary contract with SLaM and be an employee of one of the organisations forming King's Health Partners, the Academic Health Sciences Centre (AHSC) of which SLaM is a

For peer review only - http://bmjopen.bmj.com/site/about/guidelines.xhtml

member. Use of CRIS for all forms of research is logged and audited and misuse would result in disciplinary action.

- Recruiters use de-identified data on CRIS to identify potential recruits. In CRIS itself, records are identified by a locally generated pseudonym (the BRC ID). This pseudonym is linked to the EHR ID number in the building of the CRIS data repository. Research users of CRIS are unable to access the link between BRC ID and EHR ID (Figure 2), which maintains the integrity of CRIS as a de-identified database (Figure 1).
- An Oversight Committee (which is chaired by a mental health ~~service user~~patient, ~~and which includes committee members who do not use CRIS for research as well as experts on ethics, research and information governance~~) manages all access to, and monitors all use of, CRIS, and reports to the Trust Caldicott Committee (which has responsibility for ensuring the protection of patient confidentiality throughout the Trust). The committee provides operational oversight and management of CRIS – including the provision of research governance for projects using CRIS; the monitoring and regular review of the effectiveness of the CRIS security model (including the de-identification processes); oversight of the administration of CRIS, including access control and maintenance, as well as the monitoring of audit logs; provision of advice on how to use CRIS; and responses to complaints related to CRIS (including from patients). There is no quorum required for individual meetings of the committee, but membership of the Committee must include patient/service user representation, a representative of the Trust's Caldicott Guardian, Trust research and development (R&D) representation and child and adolescent mental health services (CAMHS) representation. The Oversight Committee, its structure and its function are an integral component of the CRIS data resource as ethically approved.
- The Oversight Committee uses ethical, legal and scientific criteria to adjudicate such access and use. Applications to use CRIS to identify potential recruits are considered only for projects that have specific ethics and research governance approval. (In other words, projects using the SLaM C4C model must first acquire ethics and research governance approval and then approval from the Oversight Committee.) In the UK,

Formatted: Indent: Left: 0"

Formatted: Font: (Default) Times, Not Italic

Formatted: Font: (Default) Times, 12 pt

Formatted: Font: Times

Formatted: Font: Times

For peer review only - http://bmjopen.bmj.com/site/about/guidelines.xhtml

acquiring this approval necessitates specifying how exactly patients will be recruited. This provides an additional layer of oversight, which helps to ensure that appropriate modes of approach are made to patients who have given 'consent to contact' when the topic of research is sensitive (e.g. researchers might be required to talk to the treating clinician before contacting the patient).

- The recruiter submits the pseudonymised BRC ID of potential recruits s/he wishes to contact to a trusted third party (TTP) appointed and monitored by the Oversight Committee.
- The TTP has project-specific access to the database linking the BRC IDs with the source EHR IDs. Technical specifications within the system ensure that CRIS cannot return the EHR ID of any patient who has not given consent for contact. Following reverse search, the TTP passes the EHR IDs of the potential recruits that have given consent back to the recruiter.
- The recruiter, through the SLaM EHR, is then able to identify and contact the patient to discuss participation in the project. Safeguards include a time limit on when the recruiter may contact patients about recruitment, and the requirement that recruiters inform each patient's care coordinator by email that they will be contacting that patient in a few days' time (to allow the opportunity for the care coordinator to suggest, for example, that an approach at the current time might not be advisable).
- The Oversight Committee monitors the research participation forms to ascertain if approaches for research participation or actual research participation appears excessive. What would constitute 'excessive' is an ethical, sociological and scientific question: what might be excessive for certain individual patients, or groups of patients, might not be for others; participating in multiple surveys might be adjudicated differently from participation in several clinical trials in a short time frame. The oversight committee is formalizing guidelines to adjudicate and respond to putatively 'excessive' research participation. Options for action include contacting the patient or clinical team about whether the patient continues to be willing to be approached about further research projects during the course of the current projects in which they are participating.

For peer review only - http://bmjopen.bmj.com/site/about/guidelines.xhtml

Involving and engaging stakeholders

There is increasing acknowledgement of the importance of engaging stakeholders and the public in designing ethical and technically robust procedures to guide the use of EHRs.[8 10 11 32] Mental health service users and patients made significant contributions to the development of our model. The Patient and Carer Participation Theme within the SLaM BRC took a central role in developing the model; the Oversight Committee is chaired by a mental health ~~service user~~patient; and consultation and engagement with service user/patient groups have taken place throughout, in line with our BRC's model of involving service users/~~service users~~patients at all stages of translational research.[33] An annual newsletter is sent to all persons in the recruitment database summarising key findings arising from the SLaM BRC, in addition to reminding people of contact details for the database (for example, if they wish later to withdraw). The SLaM C4C model has a dedicated website, and there are regular dissemination activities within the Trust to publicize the model. Details of how to contact the SLaM C4C team (who assist with requests to join the register, as well as provide information on how withdraw from the register) are also disseminated online and via other media. Those who consented to join the register but have been discharged from the Trust are regularly sent a reminder that they are on the register as well as information on how to withdraw if they wish. As of July 2014, 30 patients have withdrawn their consent from being listed on the register. (See Figure 4 for a summary of key features of the SLaM C4C model.)

Formatted: Font: (Default) Times, Not Italic

Formatted: Font: (Default) Times, Not Italic

Formatted: Font: (Default) Times, Not Italic

Formatted: Font: (Default) Times, Not Italic

Insert Figure 4 near here

Results

Summary statistics from the implementation of SLaM C4C

SLaM C4C started to be implemented across the South London & Maudsley NHS Trust in May 2012, and the aim is to implement C4C across the entire Trust, which serves a total of approximately 35,000 active patients, split across seven Clinical Academic Groups (CAGs). All patients are gradually being approached and asked whether they will

For peer review only

provide ‘consent for contact’. There are no exclusion criteria. Implementation commenced in services where there was already support for and/or enthusiasm about C4C. The figures from 25 October 2013 show that a total of 2,106 patients had been approached, of whom 1,560 had given consent and 546 had not: a 74.1% consent rate. Table 1 presents descriptive statistics, from October 2013, of those approached to give consent for contact, in relation to gender, ethnicity and age.

Insert Table 1 near here

Table 1: Descriptive data regarding patients approached for SLaM C4C

	N total	N consenting	Consent rate (%)
Gender			
**Male**	1078	844	78.3
**Female**	1028	716	69.6
Self-assigned Ethnicity (amalgamated)			
**White**	1228	894	72.8
**Caribbean, African or Any other Black Background**	496	360	72.6
**Not stated**	205	168	82.0
**Other**	100	78	78.0
**Indian, Pakistani, Bangladeshi or Any other Asian Background**	77	60	77.9
Age*			
**0 to 19**	841	667	79.3
**20 to 29**	182	165	90.7
**30 to 44**	232	213	91.8
**45 to 74**	370	283	76.5
**75 +**	481	232	48.2

Data captured on 25 October 2013.

* NB Age distribution is not representative of the Trust as a whole, since implementation of SLaM C4C to date has significantly focused on Mental Health of Older Adults Services, and Child and Adolescent Mental Health Services

For peer review only - http://bmjopen.bmj.com/site/about/guidelines.xhtml

Discussion

Initial data suggest that the SLaM C4C model is capable of accelerating the development of a culture of active research participation that is founded on the ethical and effective use of electronic health records. The model has been successfully developed with significant service userpatient/service user involvement, has received necessary approval and endorsement from all relevant governance bodies, and is currently resulting in almost three of every four patients approached agreeing to join the recruitment database. Public surveys and bioethical analyses commonly indicate that mental health is a sensitive domain in relation to EHRs, in light of concerns about potential stigma and discrimination.[32 34 35] The current consent figures in this secondary mental health services context are therefore encouraging vis-à-vis the model's transferability elsewhere.

Preliminary data from the implementation of SLaM C4C raise some interesting lines of investigation. That there are currently lower rates of consent from patients over 75 concurs with other research that indicates that older people are more likely to refuse participation in health research.[36] That there are currently higher rates of consent by men than women parallels other findings indicating that men are more likely to consent than women to a review of their medical records.[35] Variations in consent rates pose questions about possible selection bias in studies using this recruitment database. More research is needed to assess how many of those giving consent for contact subsequently consent to participate in particular research studies. Studies currently underway that are using the SLaM C4C model to recruit participants include: a longitudinal study to discover and validate biomarkers in Alzheimer's Disease; an interventional randomised, double blind exploratory study investigating the effects of an atypical antidepressant on cognition and BOLD fMRI signals in subjects remitted from depression and controls; a study examining differences in cognitive appraisals of anomalous experiences and different facial emotions at a cognitive and neural level between individuals with psychotic symptoms with a need for care versus those without a need for care; and a study aiming prospectively to validate a set of questionnaires for the monitoring of treatment outcomes and side-effects (including suicidality and self-harm) in general

Formatted: Font: Times, 12 pt

Formatted: Font: Times, 12 pt

Formatted: Font: Times, 12 pt

Formatted: Font: Times, 12 pt

Formatted: Font: Times, 12 pt

Formatted: Font: Times, 12 pt

Formatted: Font: Times, 12 pt

Formatted: Font: (Default) Times

For peer review only - http://bmjopen.bmj.com/site/about/guidelines.xhtml

populations and in populations known to be at elevated risk of suicide.

Formatted: Font: Times, 12 pt

Formatted: Font: Not Bold, Not Italic

The model that we have presented adheres to current best practice in recruiting patients[24], most notably as regards ensuring that no identifiable patient information is available to researchers without the patient's consent. The model appears to be effective in its implementation – both in enabling the creation of a recruitment database, and in terms of acceptability to patients. We believe the model to be generalizable to other health services contexts that employ EHRs. Our intent when designing it was to guard against the erosion of trust in research – a key risk associated with the use of medical records without consent[34] – both through adhering to consent for contact principles (i.e. ensuring that patients are explicitly asked for their consent before their medical records can be looked at for research purposes) and committing regularly to engage with members of the recruitment database about ongoing research and about their current willingness to be contacted about potential research possibilities. We designed the model to allow patients greater autonomy in decisions to take part in research, through lessening the gatekeeper role of clinicians: evidence from a prospective cohort study and a qualitative process evaluation indicates that such gatekeeper functions can impede equitable access to research.[13 37]

A new culture of research participation?

The use, linkage and further development of large EHR datasets are likely to transform relations between researchers, clinicians, patients and their data. The implementation of SLaM C4C, across a large mental health provider, generates, we believe, effectively a field site or laboratory in which to study these potential transformations. The Oversight Committee regularly captures descriptive data that give broad indications of how implementation of SLaM C4C is proceeding (both as regards patients approached, and as regards the number and type of research studies applying to use SLaM C4C). In addition, SLaM BRC is planning additional research and evaluation studies (see Figure 5 for indicative research studies).

For peer review only

For peer review only - http://bmjopen.bmj.com/site/about/guidelines.xhtml

Insert Figure 5 near here

Our BRC's ongoing collection of survey and audit data, as well as planned research studies, comprise a powerful means through which to interrogate and analyse the sociological, ethical, technical and governance-related ramifications of large-scale EHR implementation of consent for contact, in which clinicians no longer provide the primary conduit for patient participation in research.

Contributorship Statement

Formatted: Font: Bold

The idea for the manuscript was conceived by FC in conversation with RS and SL. FC drafted the manuscript, with substantial text contributions from MB, SL and RS. All authors were involved in critical revision of the manuscript before submission, and approved the final manuscript. All authors developed and/or refined the consent for contact model described in the paper. FC is the guarantor. FC is Chair of the CRIS Oversight Committee and was Chair of the Security and Confidentiality Procedures Working Group that developed the security model for CRIS; MB project managed the development of CRIS; MD directed information strategy within SLaM during the development of CRIS and SLaM C4C; MH developed the protocol for patients lacking mental capacity; MS is Head of Information Governance and the Caldicott representative at SLaM; TW is Patient and Carer Participation Theme Lead for the Biomedical Research Centre for Mental Health; SL was director of the Biomedical Research Centre for Mental Health during the development of CRIS and SLaM C4C; RS led the academic development of CRIS. RS, SL and MH secured funding for the work.

Competing Interests

For peer review only - http://bmjopen.bmj.com/site/about/guidelines.xhtml

SL is co-ordinator of the European Medical Information Framework (www.emif.eu), a public private partnership with funding from EU and pharmaceutical companies that seeks to make use of both research and routine clinical data for research.
There are no other competing interests.

Funding Sources

Authors FC, MB, -MH, TW, SL and RS – as well as CRIS itself – are or have been part-funded by the National Institute for Health Research (NIHR) Biomedical Research Centre and Dementia Biomedical Research Unit at South London and Maudsley NHS Foundation Trust and King's College London for this research.acknowledge financial support from the National Institute for Health Research (NIHR) Specialist Biomedical Research Centre for Mental Health award to the South London and Maudsley NHS Foundation Trust and the Institute of Psychiatry, King's College London. SL and TW acknowledge the NIHR for their Senior Investigator Awards. The development of the SLAM BRC Case Register was funded by a Capital Award from the UK NIHR and is further supported through the BRC Nucleus funded jointly by the Guy's and St Thomas' Trustees and South London and Maudsley Special Trustees. The funders had no role in study design, data collection and analysis, decision to publish, or preparation of the manuscript.

Formatted: Font: (Default) Times, 12 pt

Formatted: Font: (Default) Times, 12 pt

Data Sharing

Formatted: Font: Bold

Extra data are available by emailing felicity.callard@kcl.ac.ukNo additional data available.

Authors' contributions

The idea for the manuscript was conceived by FC in conversation with RS and SL. FC drafted the manuscript, with substantial text contributions from MB, SL and RS. All authors were involved in critical revision of the manuscript before submission, and approved the final manuscript. All authors developed and/or refined the consent for contact model described in the paper. FC is the guarantor. FC is Chair of the CRIS

For peer review only - http://bmjopen.bmj.com/site/about/guidelines.xhtml

Oversight Committee and was Chair of the Security and Confidentiality Procedures Working Group that developed the security model for CRIS; MB project managed the development of CRIS; MD directs information strategy within SLaM; MH developed the protocol for patients lacking mental capacity; MS is Head of Information Governance and the Caldicott representative at SLaM; TW is Patient and Carer Participation Theme Lead for the Biomedical Research Centre for Mental Health; SL is director of the Biomedical Research Centre for Mental Health; RS led the academic development of CRIS. RS, SL and MH secured funding for the work.

Acknowledgements

We thank the NIGB Ethics and Confidentiality Committee for its helpful comments on and input into the final design of the model for recruitment. We also thank the CRIS Oversight Committee and the CRIS Security & Confidentiality Procedures Working Group. Andrea Fernandes (CRIS Administrator), Jenny Liebscher (Research & Development [R&D] Governance and Delivery Manager for the SLaM and Institute of Psychiatry R&D Office), Sheri Oduola (Consent for Contact (C4C) Project Manager) and Megan Pritchard (CRIS Training and Development Lead) provided important details on how the SLaM C4C model is being implemented in practice.

Formatted: Right: 0", Space After: 0 pt, Line spacing: single, Tab stops: Not at 0.15" + 0.5"

Formatted: Font: (Default) Times, 12 pt

Formatted: Font: (Default) Times, 12 pt

Formatted: Font: (Default) Times, 12 pt

Formatted: Font: 12 pt

Competing Interests

SL is co-cordinator of the European Medical Information Framework (www.emif.eu), a public private partnership with funding from EU and pharmaceutical companies that seeks to make use of both research and routine clinical data for research.

There are no other conflicts of interest.

References

For peer review only - http://bmjopen.bmj.com/site/about/guidelines.xhtml

1. uBiome. Secondary. http://ubiome.com.
2. Higginson IJ, Evans CJ, Grande G, et al. Evaluating complex interventions in end of life care: the MORECare statement on good practice generated by a synthesis of transparent expert consultations and systematic reviews. BMC Med 2013;**11**:111 doi: 10.1186/1741-7015-11-111[published Online First: Epub Date]|.
3. Ross S, Grant A, Counsell C, et al. Barriers to participation in randomised controlled trials: a systematic review. J Clin Epidemiol 1999;**52**(12):1143-56
4. Zerhouni EA. Translational research: moving discovery to practice. Clin Pharmacol Ther 2007;**81**(1):126-8 doi: 10.1038/sj.clpt.6100029[published Online First: Epub Date]|.
5. Department of Health. The NHS Constitution for England: Department of Health, 2013.
6. Thomas R, Walport M. Data Sharing Review Report. London: Ministry of Justice, 2008.
7. Buckley BS, Murphy AW, MacFarlane AE. Public attitudes to the use in research of personal health information from general practitioners' records: a survey of the Irish general public. J Med Ethics 2011;**37**(1):50-5 doi: 10.1136/jme.2010.037903[published Online First: Epub Date]|.
8. Armstrong V, Barnett J, Cooper H, et al. Public perspectives on the governance of biomedical research: a qualitative study in a deliberative context. London: Wellcome Trust, 2007.
9. Willison DJ, Schwartz L, Abelson J, et al. Alternatives to project-specific consent for access to personal information for health research: what is the opinion of the Canadian public? J Am Med Inform Assoc 2007;**14**(6):706-12 doi: 10.1197/jamia.M2457[published Online First: Epub Date]|.
10. new economics foundation. Who sees what: exploring public views on personal electronic health records. London: new economics foundation, 2010.
11. Academy of Medical Sciences. Personal data for public good: using health information in medical research. London: The Academy of Medical Sciences, 2006.
12. Hewison J, Haines A. Overcoming barriers to recruitment in health research. BMJ 2006;**333**(7562):300-2 doi: 10.1136/bmj.333.7562.300[published Online First: Epub Date]|.
13. Knowles RL, Bull C, Wren C, et al. Ethics, governance and consent in the UK: implications for research into the longer-term outcomes of congenital heart defects. Arch Dis Child 2011;**96**(1):14-20 doi: 10.1136/adc.2008.152975[published Online First: Epub Date]|.
14. Academy of Medical Sciences. A new pathway for the regulation and governance of health research. London: The Academy of Medical Sciences, 2011.
15. Cheah S, O'Donoghue S, Daudt H, et al. Permission to contact (PTC)--a strategy to enhance patient engagement in translational research. Biopreserv Biobank 2013;**11**(4):245-52 doi: 10.1089/bio.2013.0023[published Online First: Epub Date]|.

For peer review only - http://bmjopen.bmj.com/site/about/guidelines.xhtml

16. LeBlanc J, Dee S, Braun L, et al. Impact of a Permission to Contact (PTC) platform on biobank enrollment and efficiency. Biopreserv Biobank 2013;**11**(3):144-8 doi: 10.1089/bio.2013.0004[published Online First: Epub Date]|.
17. UK Biobank. Biobank Information Leaflet, 2010.
18. Brooks CJ, Stephens JW, Price DE, et al. Use of a patient linked data warehouse to facilitate diabetes trial recruitment from primary care. Prim Care Diabetes 2009;**3**(4):245-8 doi: 10.1016/j.pcd.2009.06.004[published Online First: Epub Date]|.
19. Embi PJ, Jain A, Clark J, et al. Effect of a clinical trial alert system on physician participation in trial recruitment. Arch Intern Med 2005;**165**(19):2272-7 doi: 10.1001/archinte.165.19.2272[published Online First: Epub Date]|.
20. McGregor J, Brooks C, Chalasani P, et al. The Health Informatics Trial Enhancement Project (HITE): Using routinely collected primary care data to identify potential participants for a depression trial. Trials 2010;**11**:39 doi: 10.1186/1745-6215-11-39[published Online First: Epub Date]|.
21. Ainsworth J, Buchan I. Preserving consent-for-consent with feasibility-assessment and recruitment in clinical studies: FARSITE architecture. Stud Health Technol Inform 2009;**147**:137-48
22. Stuardi T, Cox H, Torgerson DJ. Database recruitment: a solution to poor recruitment in randomized trials? Fam Pract 2011;**28**(3):329-33 doi: 10.1093/fampra/cmq108[published Online First: Epub Date]|.
23. Nuffield Council on Bioethics. New Working Party on Biodata. Secondary New Working Party on Biodata 2013. http://www.nuffieldbioethics.org/news/new-working-party-biodata.
24. Caldicott F. Information: to share or not to share?: the information governance review. London: Department of Health, 2013.
25. Great Britain. Department of H. Information: to share or not to share?: Government response to the Caldicott Review. In: Great Britain. Department of H, ed.: Department of Health, 2013.
26. Health Research Authority. The HRA welcomes the publication of Dame Fiona Caldicott's Information Governance Review. 2013
27. Stewart R, Soremekun M, Perera G, et al. The South London and Maudsley NHS Foundation Trust Biomedical Research Centre (SLAM BRC) case register: development and descriptive data. BMC Psychiatry 2009;**9**:51 doi: 10.1186/1471-244X-9-51[published Online First: Epub Date]|.
28. Chang CK, Hayes RD, Broadbent M, et al. All-cause mortality among people with serious mental illness (SMI), substance use disorders, and depressive disorders in southeast London: a cohort study. BMC Psychiatry 2010;**10**:77 doi: 10.1186/1471-244X-10-77[published Online First: Epub Date]|.
29. Fernandes AC, Cloete D, Broadbent MT, et al. Development and evaluation of a de-identification procedure for a case register sourced from mental health electronic records. BMC Med Inform Decis Mak 2013;**13**:71 doi: 10.1186/1472-6947-13-71[published Online First: Epub Date]|.
30. Robotham D, Riches S, Perdue I, et al. Consenting for contact? Linking electronic health records to a research register within psychosis services. Under review
31. *Mental Capacity Act 2005 : code of practice*. London: TSO, 2007.

For peer review only - http://bmjopen.bmj.com/site/about/guidelines.xhtml

32. Ipsos MORI. The use of personal health information in medical research: general public consultation. Final report. London: Medical Research Council, 2007.
33. Callard F, Rose D, Wykes T. Close to the bench as well as at the bedside: involving service users in all phases of translational research. Health Expect 2012;**15**(4):389-400 doi: 10.1111/j.1369-7625.2011.00681.x[published Online First: Epub Date]|.
34. Rothstein MA. Is deidentification sufficient to protect health privacy in research? Am J Bioeth 2010;**10**(9):3-11 doi: 10.1080/15265161.2010.494215[published Online First: Epub Date]|.
35. Hill EM, Turner EL, Martin RM, et al. "Let's get the best quality research we can": public awareness and acceptance of consent to use existing data in health research: a systematic review and qualitative study. BMC Med Res Methodol 2013;**13**:72 doi: 10.1186/1471-2288-13-72[published Online First: Epub Date]|.
36. Williams B, Irvine L, McGinnis AR, et al. When "no" might not quite mean "no"; the importance of informed and meaningful non-consent: results from a survey of individuals refusing participation in a health-related research project. BMC Health Serv Res 2007;**7**:59 doi: 10.1186/1472-6963-7-59[published Online First: Epub Date]|.
37. Patterson S, Kramo K, Soteriou T, et al. The great divide: a qualitative investigation of factors influencing researcher access to potential randomised controlled trial participants in mental health settings. J Ment Health 2010;**19**(6):532-41 doi: 10.3109/09638237.2010.520367[published Online First: Epub Date]|.

For peer review only - http://bmjopen.bmj.com/site/about/guidelines.xhtml

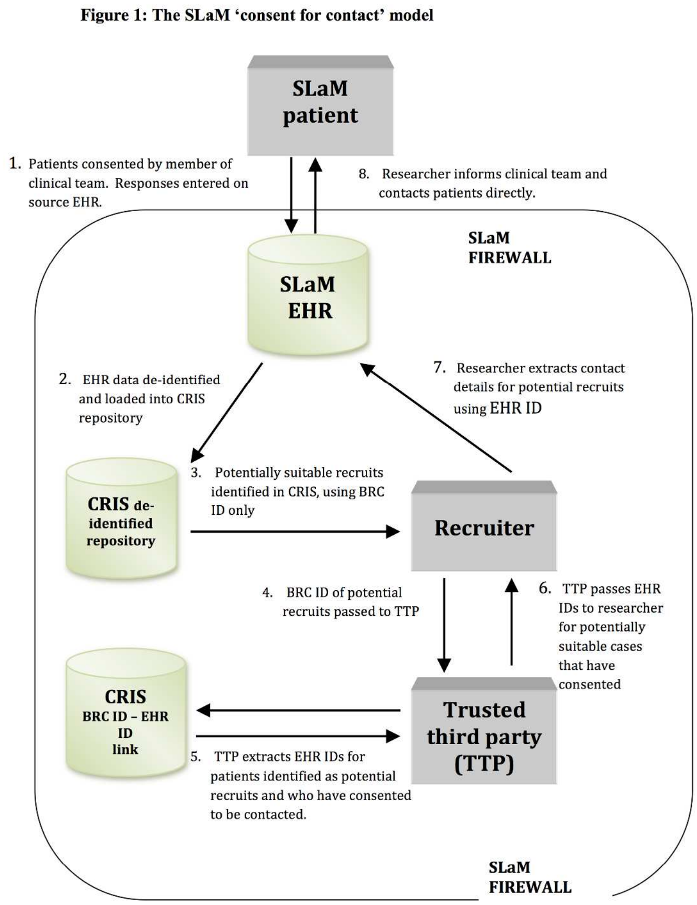

Figure 1: The SLaM 'consent for contact' model
209x297mm (300 x 300 DPI)

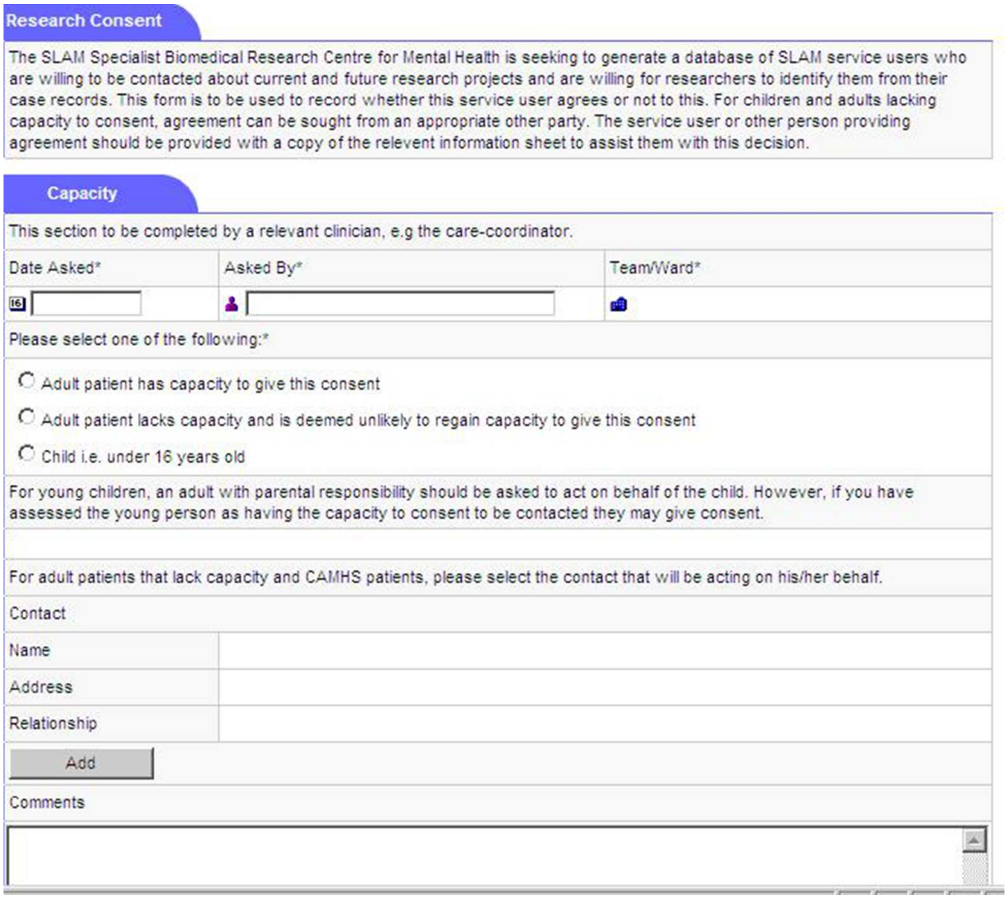

Research Consent

The SLAM Specialist Biomedical Research Centre for Mental Health is seeking to generate a database of SLAM service users who are willing to be contacted about current and future research projects and are willing for researchers to identify them from their case records. This form is to be used to record whether this service user agrees or not to this. For children and adults lacking capacity to consent, agreement can be sought from an appropriate other party. The service user or other person providing agreement should be provided with a copy of the relevent information sheet to assist them with this decision.

Capacity

This section to be completed by a relevant clinician, e.g the care-coordinator.

Date Asked*	Asked By*	Team/Ward*

Please select one of the following:*

- ○ Adult patient has capacity to give this consent
- ○ Adult patient lacks capacity and is deemed unlikely to regain capacity to give this consent
- ○ Child i.e. under 16 years old

For young children, an adult with parental responsibility should be asked to act on behalf of the child. However, if you have assessed the young person as having the capacity to consent to be contacted they may give consent.

For adult patients that lack capacity and CAMHS patients, please select the contact that will be acting on his/her behalf.

Contact

Name	
Address	
Relationship	

Add

Comments

Figure 2: Screen shot of the SLaM C4C Patient Participation Form in the original source EHR (Part i)
55x49mm (300 x 300 DPI)

only

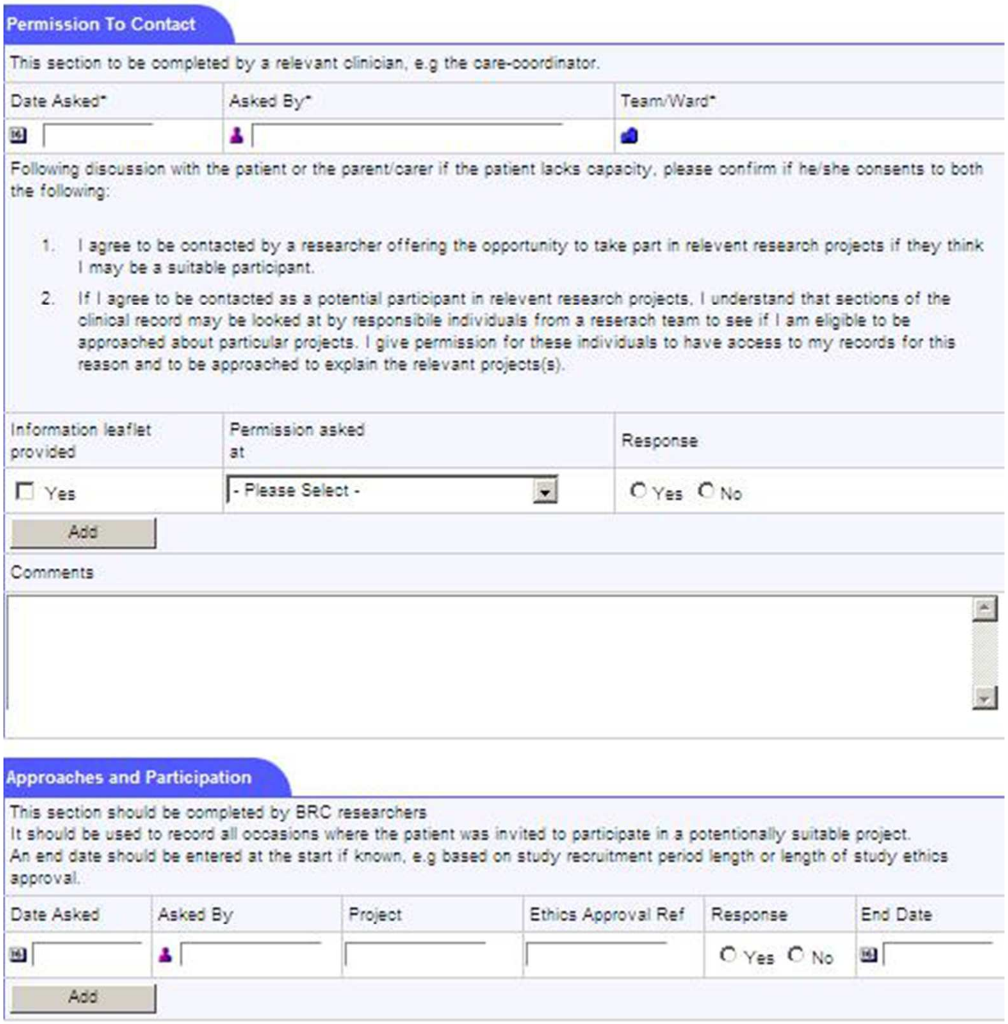

Permission To Contact

This section to be completed by a relevant clinician, e.g the care-coordinator.

Date Asked*	Asked By*	Team/Ward*

Following discussion with the patient or the parent/carer if the patient lacks capacity, please confirm if he/she consents to both the following:

1. I agree to be contacted by a researcher offering the opportunity to take part in relevent research projects if they think I may be a suitable participant.
2. If I agree to be contacted as a potential participant in relevent research projects, I understand that sections of the clinical record may be looked at by responsibile individuals from a reserach team to see if I am eligible to be approached about particular projects. I give permission for these individuals to have access to my records for this reason and to be approached to explain the relevant projects(s).

Information leaflet provided	Permission asked at	Response
Yes	- Please Select -	Yes No

Add

Comments

Approaches and Participation

This section should be completed by BRC researchers
It should be used to record all occasions where the patient was invited to participate in a potentionally suitable project.
An end date should be entered at the start if known, e.g based on study recruitment period length or length of study ethics approval.

Date Asked	Asked By	Project	Ethics Approval Ref	Response	End Date
				Yes No	

Add

Figure 2: Screen shot of the SLaM C4C Patient Participation Form in the original source EHR (part ii)
46x47mm (300 x 300 DPI)

Appendix 1a Information sheet for patients (SLaM C4C model)
Appendix 1b Information sheet for parents and guardians (SLaM C4C model)
Appendix 1c Information sheet for people responsible for another adult (SLaM C4C model)

For peer review only

South London and Maudsley NHS
NHS Foundation Trust

Participating in Research: Information for patients

Biomedical Research Centre
Clinical Record Interactive Search

We are looking for people who wish to participate in research. In order to contact you about research projects for which you might be suitable, we would like to ask your permission to review your health records at a future date.

This research is conducted within the Biomedical Research Centre, which is a partnership between South London and Maudsley NHS Foundation Trust (SLaM) and the Institute of Psychiatry at King's College London.

Our aim is to convert research findings into clear improvements in health for people with mental health and related problems. You can help us to do this. Volunteers who take part in research play a crucial role in improving the lives of thousands of people. We are creating a list of people who might be interested in taking part in current or future research projects.

If you agree to be part of this list, our researchers will be able to look at your SLaM records to see if you might be suitable for a research project (for example because of the symptoms you are experiencing or the medication you are taking). If you are potentially suitable for a project, a researcher will contact you to discuss whether you might like to participate.

We are only asking for access to your full clinical record with SLaM. We will not need to see other records.

For peer review only

What type of research is carried out at the Biomedical Research Centre for Mental Health?

We carry out a large amount of research into the causes and consequences of mental health problems, from symptoms occurring in children to those occurring at the end of life. The Biomedical Research Centre particularly focuses on finding better ways to treat mental health problems and improve the lives of those affected. Our research projects have examined common mental health disorders (like anxiety and depression), dementia, psychosis, substance use disorders, and much more. The research you might take part in could involve brain scans, puzzles, blood tests, questionnaires, interviews, computer games, or completion of a week-long diary.

For more information about our research, visit the SLaM website and search for 'BRC': http://www.slam.nhs.uk. Research is the only way to develop better treatments. Nearly all research at some point needs the help of those affected by particular health conditions in order to develop these treatments.

What will happen if I agree to be on the list?

If you agree to be on the list, this simply means that you are allowing our researchers to contact you if they think you might be suitable for a particular research project. It is possible that you may not be contacted at all, and it is possible that you will be contacted about a study which is not directly relevant to the symptoms you have been experiencing. If you agree for your name to be on this list, there is no commitment to take part in any future project.

Our researcher would approach you with information about each individual project, and would give you time to think about participating. You would then be free to agree or disagree without giving a reason for this. All we are asking for now is your permission to review your SLaM health records in the future so that our researchers can contact you about research projects for which you might be suitable.

There may not be a project suitable for you to take part in straightaway, but we will keep people who are on the list updated about the research being done through an annual newsletter.

For peer review only

Can I choose the research projects I would like to be involved in?

Yes. You can specify any types of research project you would be interested in or would prefer not to be contacted about. You can let your care coordinator know about your preferences and these can be recorded on your electronic record.

If you wish to limit the number of times in a year we approach you about research studies, your care coordinator can also include this on your record, as well as any other concerns you might have.

If you are discharged from SLaM in the future, we will ask you again about whether you wish to remain on this list, but otherwise your name will be removed automatically. A decision to withdraw at any time, or a decision not to be put on the list in the first place, will not in any way affect the standard of care you receive.

Does I have to be on the list?

No. It is up to you to decide whether you want to be on the list. This is an 'opt in' (rather than an 'opt out') system. You do not have to give a reason for not being on the list. If you decide to be on the list, your permission will be recorded on your electronic health record (i.e. on the computer system). We will give you a copy of this information for you to keep.

You are free to withdraw this agreement at any time without giving a reason. If you want to withdraw, you can ask your care coordinator to alter the health record, or contact the list administrator (contact details below).

Will my personal details be kept confidential?

We will comply with UK confidentiality laws to make sure that confidentiality is protected. Only our researchers who have gone through the necessary checks required by law can see personal information. However, when considering this, it is important that you realise that any piece of information recorded in your case record might potentially be used as a basis for contacting you in the future. If you wish to participate but do not wish a particular part of your record to be used in this way then please let your care coordinator know and they can record this preference. If you are interested in finding out more about the list or participating in any of our research you can contact the list administrator by phoning 020 3228 8553 or emailing cris.administrator@kcl.ac.uk

For peer review only

South London and Maudsley NHS
NHS Foundation Trust

Contact SLaM

SLaM switchboard: 020 3228 6000
SLaM website: www.slam.nhs.uk

Patient Advice and Liaison Service (PALS)

PALS is here to listen and support you in whatever way we can. We want your experience at SLaM to be positive. If you are not happy about something at SLaM, we will try to help you. If you decide you want to make a complaint, we can advise you how to do this.

PALS 24hr information line: 0800 731 2864
PALS website: www.slam.nhs.uk/pals
PALS email: pals@slam.nhs.uk

Travel

For the quickest way to plan your journey to a SLaM service try Transport for London's online journey planner - www.tfl.gov.uk/journeyplanner or call 020 7222 1234 for 24hr travel information.

If you would like a large print, audio, Braille or a translated version of this leaflet then please ask us.

only

KING'S HEALTH PARTNERS
Pioneering better health for all

Reference: C4C
Date of publication: 1/3/2012
Review date: 1/3/2013

For peer review only - http://bmjopen.bmj.com/site/about/guidelines.xhtml

South London and Maudsley NHS
NHS Foundation Trust

Participating in Research: Information for parents and guardians

Biomedical Research Centre
Clinical Record Interactive Search

We are looking for people who wish to participate in research. In order to contact you about research projects for which your son or daughter might be suitable, we would like to ask your permission to review their health records at a future date.

This research is conducted within the Biomedical Research Centre, which is a partnership between South London and Maudsley NHS Foundation Trust (SLaM) and the Institute of Psychiatry at King's College London.

Our aim is to convert research findings into clear improvements in health for people with mental health and related problems. You can help us to do this. Volunteers who take part in research play a crucial role in improving the lives of thousands of people. We are creating a list of people who might be interested in taking part in current or future research projects.

This includes people receiving SLaM services and we are asking you as the parent (or adult with parental responsibility) whether you agree to the inclusion of your son or daughter on this list.

If you agree for your son or daughter to be part of this list, our researchers will be able to look at their SLaM records to see if they might be suitable for a research project (for example because of the symptoms they are experiencing or the medication they are taking). If they are potentially suitable for a project, a researcher will contact you and your son or daughter to discuss whether they might like to participate.

We are only asking for access to their full clinical record at SLaM. We will not need to see other records.

For peer review only - http://bmjopen.bmj.com/site/about/guidelines.xhtml

What type of research is carried out at the Biomedical Research Centre for Mental Health?

We carry out a large amount of research into the causes and consequences of mental health problems, from symptoms occurring in children to those occurring at the end of life. The Biomedical Research Centre particularly focuses on finding better ways to treat mental health problems and improve the lives of those affected. Our research projects have examined common mental health disorders (like anxiety and depression), dementia, psychosis, substance use disorders, and much more. The research your son or daughter might take part in could involve brain scans, puzzles, blood tests, questionnaires, interviews, computer games, or completion of a week-long diary.

For more information about our research, visit the SLaM website and search for 'BRC': http://www.slam.nhs.uk. Research is the only way to develop better treatments. Nearly all research at some point needs the help of those affected by particular health conditions in order to develop these treatments.

What will happen if my son or daughter is on the list?

If you agree to your son or daughter being on the list, and if they do not object themselves, this simply means that you are both allowing our researchers to get in contact if they think your son or daughter might be suitable for a particular research project. It is possible that you may not be contacted at all, and it is possible that you will be contacted about a study which is not directly relevant to the symptoms your son or daughter has been experiencing.

If you agree to your son's or daughter's name being on this list, there is no commitment to take part in any future project. Our researcher would approach you both with information about each individual project, and would give you both time to think about participating. Both of you would then be free to agree or disagree without giving a reason for this.

All we are asking for now is your permission to review this person's SLaM health records in the future so that our researchers can make contact about research projects for which your son or daughter might be suitable.

There may not be a project suitable for your son or daughter to take part in straightaway, but we will keep people who are on the list updated about the research being done through an annual newsletter.

For peer review only

Can we choose the research projects we would like to be involved in?

Yes. Either you can specify any types of research project you will be interested in or would prefer not to be contacted about. You can let the person's care coordinator know about your preferences and these can be recorded on their electronic record.

If either of you wish to limit the number of times in a year we approach you about research studies, their care coordinator can also include this on their record, as well as any other concerns you might have.

If your son or daughter is discharged from SLaM in the future, we will ask you again about whether you wish them to remain on this list, but otherwise their name will be removed automatically. A decision to withdraw at any time, or a decision not to be put on the list in the first place, will not in any way affect the standard of care they receive.

Does my son or daughter have to be on the list?

No. It is up to both of you to decide whether you want to be on the list. This is an 'opt in' (rather than an 'opt out') system. You do not have to give a reason for not being on the list. If both of you decide to be on the list, your permission will be recorded on the person's electronic health record (i.e. on the computer system). We will give you a copy of this information for you to keep.

Either of you are free to withdraw this agreement at any time without giving a reason. If either of you want to withdraw, you can ask your son's or daughter's care coordinator to alter the health record, or contact the list administrator (contact details below).

Will personal details be kept confidential?

We will comply with UK confidentiality laws to make sure that confidentiality is protected. Only our researchers who have gone through the necessary checks required by law can see personal information. However, when considering this, it is important that you realise that any piece of information recorded in you son's or daughter's case record might potentially be used as a basis for making contact in the future. If you wish to participate but do not wish a particular part of their record to be used in this way then please let their care coordinator know and they can record this preference. If you are interested in finding out more about the list or participating in any of our research you can contact the list administrator by phoning 020 3228 8553 or emailing cris.administrator@kcl.ac.uk

For peer review only

South London and Maudsley NHS
NHS Foundation Trust

Contact SLaM

SLaM switchboard: 020 3228 6000
SLaM website: www.slam.nhs.uk

Patient Advice and Liaison Service (PALS)

PALS is here to listen and support you in whatever way we can. We want your experience at SLaM to be positive. If you are not happy about something at SLaM, we will try to help you. If you decide you want to make a complaint, we can advise you how to do this.

PALS 24hr information line: 0800 731 2864
PALS website: www.slam.nhs.uk/pals
PALS email: pals@slam.nhs.uk

Travel

For the quickest way to plan your journey to a SLaM service try Transport for London's online journey planner - www.tfl.gov.uk/journeyplanner or call 020 7222 1234 for 24hr travel information.

If you would like a large print, audio, Braille or a translated version of this leaflet then please ask us.

KING'S HEALTH PARTNERS

Reference: C4C
Date of publication: 1/3/2012
Review date: 1/3/2013

Pioneering better health for all

For peer review only - http://bmjopen.bmj.com/site/about/guidelines.xhtml

South London and Maudsley NHS
NHS Foundation Trust

Participating in Research: Information for people responsible for another adult

Biomedical Research Centre
Clinical Record Interactive Search

We are looking for people who wish to participate in research. In order to contact you about research projects for which the person you are responsible for might be suitable, we would like to ask your permission to review their health records at a future date.

This research is conducted within the Biomedical Research Centre, which is a partnership between South London and Maudsley NHS Foundation Trust (SLaM) and the Institute of Psychiatry at King's College London.

Our aim is to convert research findings into clear improvements in health for people with mental health and related problems. You can help us to do this. Volunteers who take part in research play a crucial role in improving the lives of thousands of people. We are creating a list of people who might be interested in taking part in current or future research projects.

This includes people receiving SLaM services and we are asking you as someone with appropriate responsibility whether you agree with the inclusion of the person you are responsible for on this list because it is felt that they are unable to make the decision themselves.

If you agree for this person to be part of this list, our researchers will be able to look at their SLaM records to see if they might be suitable for a research project (for example because of the symptoms they are experiencing or the medication they are taking). If they are potentially suitable for a project, a researcher will contact you and the person to discuss whether they might like to participate.

We are only asking for access to their full clinical record at SLaM. We will not need to see other records.

For peer review only

What type of research is carried out at the Biomedical Research Centre for Mental Health?

We carry out a large amount of research into the causes and consequences of mental health problems, from symptoms occurring in children to those occurring at the end of life. The Biomedical Research Centre particularly focuses on finding better ways to treat mental health problems and improve the lives of those affected. Our research projects have examined common mental health disorders (like anxiety and depression), dementia, psychosis, substance use disorders, and much more. The research the person might take part in could involve brain scans, puzzles, blood tests, questionnaires, interviews, computer games, or completion of a week-long diary.

For more information about our research, visit the SLaM website and search for 'BRC': http://www.slam.nhs.uk. Research is the only way to develop better treatments. Nearly all research at some point needs the help of those affected by particular health conditions in order to develop these treatments.

What will happen if I agree for this person to be on the list?

If you agree to this person being on the list, and if they do not object themselves, this simply means that you are both allowing our researchers to get in contact if they think this person might be suitable for a particular research project. It is possible that you may not be contacted at all, and it is possible that you will be contacted about a study which is not directly relevant to the symptoms they have been experiencing.

If you agree to the person's name being on this list, there is no commitment to take part in any future project. Our researcher would approach you both with information about each individual project, and would give you both time to think about participating. Both of you would then be free to agree or disagree without giving a reason for this.

All we are asking for now is your permission to review this person's SLaM health records in the future so that our researchers can make contact about research projects for which this person might be suitable.

There may not be a project suitable for this person to take part in straightaway, but we will keep people who are on the list updated about the research being done through an annual newsletter.

For peer review only

Can we choose the research projects we would like to be involved in?

Yes. Either you can specify any types of research project you will be interested in or would prefer not to be contacted about. You can let the person's care coordinator know about your preferences and these can be recorded on their electronic record.

If either of you wish to limit the number of times in a year we approach you about research studies, their care coordinator can also include this on their record, as well as any other concerns you might have.

If this person is discharged from SLaM in the future, we will ask you again about whether you wish them to remain on this list, but otherwise their name will be removed automatically. A decision to withdraw at any time, or a decision not to be put on the list in the first place, will not in any way affect the standard of care they receive.

Does this person have to be on the list?

No. It is up to both of you to decide whether you want to be on the list. This is an 'opt in' (rather than an 'opt out') system. You do not have to give a reason for not being on the list. If both of you decide to be on the list, your permission will be recorded on the person's electronic health record (i.e. on the computer system). We will give you a copy of this information for you to keep.

Either of you are free to withdraw this agreement at any time without giving a reason. If either of you want to withdraw, you can ask the person's care coordinator to alter the health record, or contact the list administrator (contact details below).

Will personal details be kept confidential?

We will comply with UK confidentiality laws to make sure that confidentiality is protected. Only our researchers who have gone through the necessary checks required by law can see personal information. However, when considering this, it is important that you realise that any piece of information recorded in the person's case record might potentially be used as a basis for making contact in the future. If you wish to participate but do not wish a particular part of their record to be used in this way then please let their care coordinator know and they can record this preference. If you are interested in finding out more about the list or participating in any of our research you can contact the list administrator by phoning 020 3228 8553 or emailing cris.administrator@kcl.ac.uk

For peer review only

South London and Maudsley NHS
NHS Foundation Trust

Contact SLaM

SLaM switchboard: 020 3228 6000
SLaM website: www.slam.nhs.uk

Patient Advice and Liaison Service (PALS)

PALS is here to listen and support you in whatever way we can. We want your experience at SLaM to be positive. If you are not happy about something at SLaM, we will try to help you. If you decide you want to make a complaint, we can advise you how to do this.

PALS 24hr information line: 0800 731 2864
PALS website: www.slam.nhs.uk/pals
PALS email: pals@slam.nhs.uk

Travel

For the quickest way to plan your journey to a SLaM service try Transport for London's online journey planner - www.tfl.gov.uk/journeyplanner or call 020 7222 1234 for 24hr travel information.

If you would like a large print, audio, Braille or a translated version of this leaflet then please ask us.

KING'S HEALTH PARTNERS

Reference: C4C
Date of publication: 1/3/2012
Review date: 1/3/2013

Pioneering better health for all

For peer review only - http://bmjopen.bmj.com/site/about/guidelines.xhtml

STROBE 2007 (v4) checklist of items to be included in reports of observational studies in epidemiology*

Checklist for cohort, case-control, and cross-sectional studies (combined)

Section/Topic	Item #	Recommendation	Reported on page #
**Title and abstract**	1	(*a*) Indicate the study's design with a commonly used term in the title or the abstract	2
		(*b*) Provide in the abstract an informative and balanced summary of what was done and what was found	3
**Introduction**			
Background/rationale	2	Explain the scientific background and rationale for the investigation being reported	5-7
Objectives	3	State specific objectives, including any pre-specified hypotheses	7
**Methods**			
Study design	4	Present key elements of study design early in the paper	8-9
Setting	5	Describe the setting, locations, and relevant dates, including periods of recruitment, exposure, follow-up, and data collection	8
Participants	6	(*a*) *Cohort study*—Give the eligibility criteria, and the sources and methods of selection of participants. Describe methods of follow-up<br>*Case-control study*—Give the eligibility criteria, and the sources and methods of case ascertainment and control selection. Give the rationale for the choice of cases and controls<br>*Cross-sectional study*—Give the eligibility criteria, and the sources and methods of selection of participants	9, 14
		(*b*) *Cohort study*—For matched studies, give matching criteria and number of exposed and unexposed<br>*Case-control study*—For matched studies, give matching criteria and the number of controls per case	
Variables	7	Clearly define all outcomes, exposures, predictors, potential confounders, and effect modifiers. Give diagnostic criteria, if applicable	14
Data sources/ measurement	8*	For each variable of interest, give sources of data and details of methods of assessment (measurement). Describe comparability of assessment methods if there is more than one group	14
Bias	9	Describe any efforts to address potential sources of bias	n/a
Study size	10	Explain how the study size was arrived at	n/a
Quantitative variables	11	Explain how quantitative variables were handled in the analyses. If applicable, describe which groupings were chosen and why	n/a descriptive statistics only
Statistical methods	12	(*a*) Describe all statistical methods, including those used to control for confounding	n/a descriptive statistics only
		(*b*) Describe any methods used to examine subgroups and interactions	n/a descriptive statistics only
		(*c*) Explain how missing data were addressed	n/a descriptive

For peer review only

			statistics only
		(*d*) *Cohort study*—If applicable, explain how loss to follow-up was addressed<br>*Case-control study*—If applicable, explain how matching of cases and controls was addressed<br>*Cross-sectional study*—If applicable, describe analytical methods taking account of sampling strategy	n/a descriptive statistics only
		(*e*) Describe any sensitivity analyses	n/a descriptive statistics only
**Results**			
Participants	13*	(a) Report numbers of individuals at each stage of study—eg numbers potentially eligible, examined for eligibility, confirmed eligible, included in the study, completing follow-up, and analysed	
		(b) Give reasons for non-participation at each stage	
		(c) Consider use of a flow diagram	
Descriptive data	14*	(a) Give characteristics of study participants (eg demographic, clinical, social) and information on exposures and potential confounders	14
		(b) Indicate number of participants with missing data for each variable of interest	14
		(c) *Cohort study*—Summarise follow-up time (eg, average and total amount)	
Outcome data	15*	*Cohort study*—Report numbers of outcome events or summary measures over time	
		Case-control study—Report numbers in each exposure category, or summary measures of exposure	
		Cross-sectional study—Report numbers of outcome events or summary measures	
Main results	16	(*a*) Give unadjusted estimates and, if applicable, confounder-adjusted estimates and their precision (eg, 95% confidence interval). Make clear which confounders were adjusted for and why they were included	
		(*b*) Report category boundaries when continuous variables were categorized	
		(*c*) If relevant, consider translating estimates of relative risk into absolute risk for a meaningful time period	
Other analyses	17	Report other analyses done—eg analyses of subgroups and interactions, and sensitivity analyses	n/a
**Discussion**			
Key results	18	Summarise key results with reference to study objectives	14
Limitations	19	Discuss limitations of the study, taking into account sources of potential bias or imprecision. Discuss both direction and magnitude of any potential bias	15
Interpretation	20	Give a cautious overall interpretation of results considering objectives, limitations, multiplicity of analyses, results from similar studies, and other relevant evidence	15-16
Generalisability	21	Discuss the generalisability (external validity) of the study results	15-16
**Other information**			
Funding	22	Give the source of funding and the role of the funders for the present study and, if applicable, for the original study on which the present article is based	17

For peer review only

*Give information separately for cases and controls in case-control studies and, if applicable, for exposed and unexposed groups in cohort and cross-sectional studies.

Note: An Explanation and Elaboration article discusses each checklist item and gives methodological background and published examples of transparent reporting. The STROBE checklist is best used in conjunction with this article (freely available on the Web sites of PLoS Medicine at http://www.plosmedicine.org/, Annals of Internal Medicine at http://www.annals.org/, and Epidemiology at http://www.epidem.com/). Information on the STROBE Initiative is available at www.strobe-statement.org.

For peer review only
